# Organelle Phylogenomics and Extensive Conflicting Phylogenetic Signals in the Monocot Order Poales

**DOI:** 10.3389/fpls.2021.824672

**Published:** 2022-01-31

**Authors:** Hong Wu, Jun-Bo Yang, Jing-Xia Liu, De-Zhu Li, Peng-Fei Ma

**Affiliations:** ^1^Germplasm Bank of Wild Species, Kunming Institute of Botany, Chinese Academy of Sciences, Kunming, China; ^2^University of Chinese Academy of Sciences, Beijing, China

**Keywords:** Poales, phylogenomic conflict, plastome, mitochondrial, nuclear

## Abstract

The Poales is one of the largest orders of flowering plants with significant economic and ecological values. Reconstructing the phylogeny of the Poales is important for understanding its evolutionary history that forms the basis for biological studies. However, due to sparse taxon sampling and limited molecular data, previous studies have resulted in a variety of contradictory topologies. In particular, there are three nodes surrounded by incongruence: the phylogenetic ambiguity near the root of the Poales tree, the sister family of Poaceae, and the delimitation of the xyrid clade. We conducted a comprehensive sampling and reconstructed the phylogenetic tree using plastid and mitochondrial genomic data from 91 to 66 taxa, respectively, representing all the 16 families of Poales. Our analyses support the finding of Bromeliaceae and Typhaceae as the earliest diverging groups within the Poales while having phylogenetic relationships with the polytomy. The clade of Ecdeiocoleaceae and Joinvilleaceae is recovered as the sister group of Poaceae. The three families, Mayacaceae, Eriocaulaceae, and Xyridaceae, of the xyrid assembly diverged successively along the backbone of the Poales phylogeny, and thus this assembly is paraphyletic. Surprisingly, we find substantial phylogenetic conflicts within the plastid genomes of the Poales, as well as among the plastid, mitochondrial, and nuclear data. These conflicts suggest that the Poales could have a complicated evolutionary history, such as rapid radiation and polyploidy, particularly allopolyploidy through hybridization. In sum, our study presents a new perspicacity into the complex phylogenetic relationships and the underlying phylogenetic conflicts within the Poales.

## Introduction

The order, Poales is a large group of flowering plants in the monocotyledons and belongs to the Commelinid clade, which includes the other three orders of Arecales, Commelinales, and Zingiberales (Angiosperm Phylogeny Group IV [APG IV] et al., 2016). With more than 20,000 species, Poales accounts for about 7% of the angiosperm and 33% of the monocot diversity, respectively (Givnish et al., 2010; Bouchenak-Khelladi et al., 2014; Alves et al., 2015). The species diversity of Poales is extremely uneven among these families. The largest family is Poaceae having about 12,000 species and the smallest one is Ecdeiocoleaceae with only three species (Christenhusz and Byng, 2016; Hochbach et al., 2018). These species are widely distributed around the world, from the equator to the pole, from floating aquatic plants to the most water-deficient deserts, and most soil types (Stevens, 2001 onward). Moreover, they are generally becoming the dominant species in their ecological communities, such as the grasses (Poaceae) in the savanna and grassland and sedges (Cyperaceae) in the wetland (Linder and Rudall, 2005). Many species of Poales also have significant economic values with Poaceae as the most economically important family in the plant kingdom (Vallée et al., 2016). This family includes many food crops, e.g., rice (*Oryza sativa* L.), wheat (*Triticum aestivum* L.), and maize (*Zea mays* L.), as well as a variety of bamboos that have multiple applications (Saarela et al., 2018). The pineapple [*Ananas comosus* (L.) Merr.] in Bromeliaceae is a famous tropical fruit and an ornamental plant (Chen et al., 2019). *Typha orientalis* C. Presl and *T. angustifolia* L. from Typhaceae are widely used in weaving and paper industries (Sun, 1992).

Based on a phylogeny using the plastid *rbc*L gene, Duvall et al. (1993) first proposed a composition of 16 families of Poales. Since then, the delineation of Poales has gradually been transformed based on the combined morphological and plastid DNA evidence (Kellogg and Linder, 1995; Angiosperm Phylogeny Group [APG], 1998; Chase et al., 2000; Bremer, 2002). In APG IV, two families, Anarthriaceae and Centrolepidaceae were merged into Restionaceae (Angiosperm Phylogeny Group IV [APG IV] et al., 2016). However, the phylogenetic relationships among them are disputed and the delimitation of the Restionaceae is still problematic (Linder and Rudall, 1993; Bremer, 2002; Michelangeli et al., 2003; Briggs et al., 2014; Hochbach et al., 2018).

The 16 families of Poales can be generally divided into five clades or grades (Linder and Rudall, 2005). The Bromeliaceae, Rapateaceae, and Typhaceae comprise the early diverging grade. The remaining four clades are called “core Poales,” which include the cyperid, xyrid, restiid, and graminid clades. The cyperid clade is strongly supported, including Cyperaceae, Juncaceae, and Thurniaceae. As the probable sister group of the cyperid clade, the xyrid clade consists of Eriocaulaceae, Mayacaceae, and Xyridaceae. However, the phylogenetic position and the relationship of the xyrid clade are still ambiguous (Givnish et al., 2018; Hochbach et al., 2018). The Restionaceae, Centrolepidaceae, and Anarthriaceae form the restiid clade and sister to the graminid clade, which encompasses the remaining four families, Ecdeiocoleaceae, Flagellariaceae, Joinvilleaceae, and Poaceae (Hochbach et al., 2018).

The phylogeny of Commelinid has always been a hot topic in the tree of life of monocots and the position of Poales in it has been determined (Barrett et al., 2016). However, a few studies focused on the Poales despite their high ecological and economical significance. The first study focusing on the Poales with a large-scale dataset was provided by Givnish et al. (2010) who sequenced 81 plastid genes of 34 representative species from 15 families. Although the backbone phylogeny of Poales has been reconstructed in this study, there are still many uncertainties about its phylogenetic relationships. First, in contrast to the earliest divergence of Bromeliaceae suggested by Chase et al. (2006),Givnish et al. (2010), Barrett et al. (2016), Givnish et al. (2018), and Hochbach et al. (2018) used the *mat*K to reveal the Bromeliaceae and Typhaceae in an early diverging polytomy and this was supported by a large number of plastid-, mitochondrial- and nuclear-based studies in which Bromeliaceae + Typhaceae were resolved as the early diverging group (Christin et al., 2008; Soltis et al., 2011; Bouchenak-Khelladi et al., 2014; Hertweck et al., 2015; Baker et al., 2021). Moreover, McKain et al. (2016) used the transcriptome data to show that Typhaceae was the first lineage to diverge within the Poales followed by Bromeliaceae. Interestingly, Darshetkar et al. (2019) analyzed the 81 plastid genes dataset like Givnish et al. (2010) with different software and models and obtained the conflicting result with Typhaceae being the first diverging lineage followed by Bromeliaceae. Second, many studies supported Ecdeiocoleaceae as sister to Poaceae (Givnish et al., 2010, 2018; Barrett et al., 2016; Darshetkar et al., 2019; Li et al., 2019). However, the Ecdeiocoleaceae + Joinvilleaceae clade was revealed to be a sister to Poaceae with increased taxon sampling (Bouchenak-Khelladi et al., 2014; McKain et al., 2016; Hochbach et al., 2018; Baker et al., 2021). Third, the relationship involving the Mayacaceae and the xyrid clade is enigmatic. Earlier studies suggested that the Mayacaceae was either within or closely related to the xyrid clade (Michelangeli et al., 2003; Linder and Rudall, 2005; Givnish et al., 2010, 2018; Darshetkar et al., 2019), but other studies suggested that it was within the cyperid clade (Chase et al., 2000, 2006; Janssen and Bremer, 2004) or between the xyrid clade and the cyperid clade (Davis et al., 2004; Hertweck et al., 2015; McKain et al., 2016). Recently, Baker et al. (2021) used 353 nuclear genes to construct the tree of life of angiosperms and found that the Mayacaceae was resolved as an early diverging lineage of Poales, only after the divergence of Bromeliaceae + Typhaceae. In short, these conflicting phylogenetic relationships may be due to the different molecular markers and/or the sparse taxon sampling, as well as various phylogenetic reconstruction methods used in previous studies.

Phylogenomics is an effective way to reconstruct the tree of life based on the genome-scale data (Delsuc et al., 2005; Yu and Zhang, 2006; Zou and Ge, 2008; Zeng et al., 2014; Wen et al., 2015). The data sources of phylogenomics can be either from organelle genomes or from nuclear genomes. In plants, organelle genomes include the plastid genome and mitochondrial genome, which mostly follow a uniparental inheritance (Zeng et al., 2014). In general, the nuclear genome has a faster evolutionary rate and the mitochondrial genome has a slower evolutionary rate while the plastid genome has a moderate rate (Tian and Li, 2002; Wei et al., 2005). The plastid genome is widely used for plant phylogenomic studies (Wei et al., 2005; Leseberg and Duvall, 2009; Gao et al., 2010; Davis et al., 2014; Zeng et al., 2014; Zhao et al., 2021). Plastid phylogenomics has successfully solved a number of plant phylogenetic problems involving taxonomic categories from high to low, e.g., the tree of life of angiosperms at the ordinal level (Li et al., 2019), phylogenetic relationships at the familial level of Malpighiales (Xi et al., 2012), or within the families of Rosaceae and Leguminosae (Zhang et al., 2017, Zhang R. et al., 2020), and the establishment of the phylogenetic framework of the bamboo tribe, Arundinarieae (Ma et al., 2014, 2017). Compared with the plastid genome, the plant mitochondrial genome has a slower evolutionary rate but a higher genomic rearrangement rate and thus poses challenges for assembly (Wei et al., 2005). These features restrict its application in the study of plant phylogeny. However, some studies have shown that mitochondrial genes could provide additional evolutionary information and are useful in reconstructing the plant phylogeny (Norman and Gray, 2001; Perrotta et al., 2002; Guo and Ge, 2004; Qiu et al., 2010; Bock et al., 2014; Sun et al., 2015; Folk et al., 2016).

In phylogenomics, hundreds to thousands of DNA loci are used, and the phylogenetic discordance or conflicting gene trees appear frequently. The reasons could be the stochastic error and the systematical error, and more often, biological factors including horizontal gene transfer, hybridization, introgression, gene duplication and loss, incomplete lineage sorting, and non-allelic gene conversion (Zou and Ge, 2008; Degnan and Rosenberg, 2009; Sun et al., 2015; Harpak et al., 2017; Kapli et al., 2020). A lot of studies have revealed inconsistencies among plastid, mitochondria, and nuclear phylogenies in plants (Wendel et al., 1995; Sun et al., 2015; Vargas et al., 2017; Hochbach et al., 2018; Jost et al., 2021). In addition, several recent studies suggested a phylogenetic discordance within the plastome at varied evolutionary scales (Gonçalves et al., 2019; Walker et al., 2019; Zhang R. et al., 2020; Yang et al., 2021), questioning the traditional concept of plastomes as a single inherited unit, or at least treating them uncritically in phylogenetic analyses (Gonçalves et al., 2019). More empirical studies are demanded to describe and understand the source, scope, and consequences of conflicting phylogenetic signals in the plastid genome (Yang et al., 2021).

Here, we adopted the classification of 16 families of Poales in this study as the basis for analyses. We reconstructed the phylogeny of Poales with plastid and mitochondrial genomes with at least two taxa or samples for each of the 16 families. The aims of this study are to (1) resolve phylogenetic relationships of key nodes for Poales involving the early diverging grade, xyrid clade, and the sister group of Poaceae; (2) explore the potential conflict within the plastid genome in the Poales phylogeny; and (3) compare the phylogenies built from different plant genomes. With the broadest taxon sampling and the genomic-scale level data, our study resolved ambiguous phylogenetic relationships within the Poales and provided new insights into the conflicting signals among different genomes.

## Materials and Methods

### Plant Materials, Sequencing, Assembly, and Annotation

We selected and sampled representative species in considering the total species of each family of the Poales. All 16 families had a sampling of at least two species. We increased samplings for large families accordingly and 19 species for both Poaceae and Cyperaceae, eight species for Bromeliaceae, four to six species for five families with a total number of 50–1,000 species, and two to four species for eight families less than 50 species. Our sampling could represent the species diversity and phylogenetic diversity of families as far as possible. For the two main systematic problems concerned, we increased the sampling of Bromeliaceae, Typhaceae, Ecodeiocoleaceae, and Joinvilleaceae by two to three species (or individuals).

Illumina sequencing of genomic DNA was undertaken with about 2–10 GB of raw data with 150 bp paired-end reads generated for each sample. Plastomes and mitochondria gene sequences were assembled *de novo* using the GetOrangelle pipeline (Jin et al., 2020). For mitochondria gene sequence, we selected a reference mitochondria genome (*Oryza sativa* L. Indica Group, NC_007886) to blast and retrieve the output contigs by GetOrangelle, many of which were derived from the mitochondrial genome, and further assembled them in Geneious v9.1.4 (Kearse et al., 2012). We also downloaded 33 published plastomes of Poales for analyses, totaling 99 Poales accessions representing 91 species from 50 genera and 16 families. Meanwhile, we selected seven species of Commelinales and Zingiberales as outgroup taxa based on previous studies (Barrett et al., 2016). The corresponding species voucher and GenBank accession numbers are listed in Supplementary Table 1.

For the mitochondrial dataset, we obtained sequences of 48 taxa (50 accessions) and downloaded 15 additional complete mitochondrial genomes. As the combination of multi-locus data of representative taxa with single loci from multiple species can generate reliable higher-level phylogenies (Talavera et al., 2021), the *cob* gene sequences of seven species were also included, resulting in a total of 66 species (72 accessions) representing 16 families and 43 genera of Poales. Since there are no published mitochondrial genomes available for the Commelinales and Zingiberales, we selected two species of Arecales [*Phoenix dactylifera* L. (NC_016740) and *Cocos nucifera* L. (NC_031696)] and one species of Asparagales [(*Allium cepa* L. (NC_030100)] as the outgroup. The corresponding species voucher and GenBank accession numbers are listed in Supplementary Table 2.

The assembled plastomes were annotated with the PGA software (Qu et al., 2019), followed by manual examination and adjustment in Geneious v9.1.4 (Kearse et al., 2012). Mitochondrial genes were annotated in Geneious v9.1.4 (Kearse et al., 2012) according to the gene annotation information of three grass mitochondrial genomes [*Oryza sativa* Indica Group, NC_007886, *Sorghum bicolor* (L.) Moench, NC_008360 and *Zea mays* L. NB, NC_007982], and mitochondrial genes with more than 80% similarity with reference sequence genome were selected for annotation and tree construction.

### Phylogenomic Analysis

We extracted the coding regions in PhyloSuite (Zhang D. et al., 2020), including 80 protein-coding, 4 rRNA and 30 tRNA genes of plastomes, and 28 mitochondrial genes, respectively. We obtained two plastid matrices, 114 genes (114PG), 80 protein-coding genes (80PG), and one mitochondrial matrix of 28 genes (28MG). The nucleotides were first translated into amino acid sequences and aligned with MAFFT v.5 (Katoh et al., 2005) software, and then we used PAL2NAL (Suyama et al., 2006) to obtain the corresponding nucleotide alignment. The ambiguously aligned regions were deleted by Gblocks (Castresana, 2000), and the parameters of allowed gap positions included all, with half, and none for the above three matrices. Matrices of the 1st + 2nd codon positions of 114PG, and 1st + 2nd of 80PG matrix, as well as the 3rd codon positions, were, respectively, obtained by SEAVIEW (Gouy et al., 2009). In total, we acquired 11 plastid matrices and four mitochondrial matrices for concatenate and coalescent analyses (Table 1).

**TABLE 1:** Characteristics of plastid and mitochondrial matrices used for phylogenetic analyses of Poales.

Matrix	No. of species	No. of genes	Length (bp)	No. of parsimony-informative sites	No. of variable sites	Missing data
114PG	106	114	83,266	31,490	8,233	12.70%
114PG-all	106	114	74,643	30,499	7,231	9.90%
114PG-half	106	114	71,445	29,436	6,832	9.30%
114PG-no	106	105	34,176	13,395	2,887	12.70%
114PG-12	106	114	58,068	22,695	5,878	0.00%
80PG	106	80	75,593	30,287	7,495	13.80%
80PG-all	106	80	67,728	29,349	6,548	10.80%
80PG-half	106	80	64,968	28,407	6,220	10.20%
80PG-no	106	72	31,115	12,908	2,494	1.90%
80PG-12	106	80	50,396	21,492	5,140	13.80%
80PG-3	106	80	25,198	8,795	2,355	13.80%
28MG	75	28	23,709	6,608	2,571	26.40%
28MG-all	75	28	22,383	6,324	2,452	25.40%
28MG-half	75	28	21,704	6,186	2,374	24.90%
28MG-no	75	28	14,529	3,624	1,478	25.30%
80PG_OR_EJ12	106	79	66,179	27,894	6,049	9.70%
80PG-all_OR_EJ12	106	79	60,600	27,135	5,443	7.50%
80PG-half_OR_EJ12	106	79	64,794	28,401	6,219	10.00%
80PG-no_OR_EJ12	106	71	29,775	12,168	2,382	9.70%
80PG_OR_BT123	106	78	74,600	29,986	7,439	14.00%
80PG-all_OR_BT123	106	78	66,735	29,048	6,492	11.00%
80PG-half_OR_BT123	106	78	63,984	28,107	6,164	10.30%
80PG-no_OR_BT123	106	71	30,921	12,857	2,483	2.00%
80PG_OR_EMX123	106	78	58,013	24,020	4,888	2.60%
80PG-all_OR_EMX123	106	78	54,738	23,602	4,647	2.20%
80PG-half_OR_EMX123	106	79	59,706	25,120	5,502	6.10%
80PG-no_OR_EMX123	106	70	27,972	11,895	2,286	2.00%

*PG, plastid gene; MG, mitochondrial gene; all, using Gblocks that allow gap positions with all; half, using Gblocks that allow gap positions with a half; no, using Gblocks that allow gap positions with none; −12, 1st + 2nd codon positions of the matrix; −3, 3rd codon; OR, outlier removed.*

For concatenate method, Bayesian inference (BI), maximum likelihood (ML), and maximum parsimony (MP) were employed. ML analyses were conducted in IQ-TREE v.1.6.10 (Nguyen et al., 2015) and RAxML v.8.2.12 (Stamatakis, 2015), respectively. IQ-TREE was performed with Ultrafast bootstrap with 1,000 replicates and the best model (Supplementary Table 3), and other default parameters. RAxML was implemented with 1,000 replicates using the GTRGAMMA model and other default parameters. MP analyses were conducted by PAUP*4.0b10 (Cummings, 2004). A heuristic search was executed with 1,000 replicates, random addition, and the tree bisection-reconnection (TBR) branch swapping with the MULTrees option. The bootstrap (BS) method with a heuristic search was performed with 1,000 replicates. BI analyses were implemented using the Mrbayes 3.6.2 (Ronquist et al., 2012) plugin in PhyloSuite (Zhang D. et al., 2020) and the best model with Corrected Akaike information criterion (AICc) was selected by ModelFinder (Kalyaanamoorthy et al., 2017). The Markov chain Monte Carlo (MCMC) algorithm was performed with 2,000,000 (plastid matrices) and 6,000,000 (mitochondrial matrix) generations. Every 1,000 generations sampled one tree with the first 25% of generations abandoned as burnt-in. The trees that remained after reaching a stationary state with the average standard deviation of the split frequencies less than 0.01 were recognized as the consensus trees.

For coalescent analysis, we used ASTRAL-III (Zhang et al., 2018) to infer the species tree with the 80 (80PG matrix) and 72 (80PG-no matrix) plastid gene trees estimated from RAxML with 100 replicates. The branches with BS less than 10% in the gene tree were collapsed using the “nw_ed” code of utilities tool (Junier and Zdobnov, 2010). FigTree v. 1.4.4 was used to visualize the phylogenetic trees (Rambaut, 2012). In general, we defined full support as the posterior probability (PP) = 1.00, BS values = 100%, and local posterior probability (LPP) = 1.00; strong support as PP ≥ 0.99, BS ≥ 85%, LPP ≥ 0.9; moderate support as 0.9 ≤ PP < 0.99, 70% ≤ BS < 85%, and 0.85 ≤ LPP < 0.9; and weak support as PP < 0.9, BS < 70%, and LPP < 0.85.

### Quantification Branch Support Values

To further quantify branch support values, we used the Quartet Sampling (QS) method with 1,000 replicates (Pease et al., 2018). This method can also distinguish branches with low information from those with multiple highly supported but mutually exclusive phylogenetic relationships. Three QS scores were used: (1) Quartet concordance (QC) is the frequency of the concordant quartet inferred over both discordant quartets. (2) Quartet differential (QD) indicates that whether one alternative relationship is sampled more often than the other. (3) Quartet informativeness (QI) is the proportion of replicates that were informative (Pease et al., 2018). The quartet sampling outputs were visualized by the R-script QS_visualization.^1^

### Quantification of Phylogenetic Signal for Alternative Tree Topologies

We assessed the phylogenetic signals for three sets of conflicting topologies based on previous studies (Shen et al., 2017, 2021; Zhang R. et al., 2020; Yang et al., 2021). Briefly, we computed the site-wise log-likelihood (SLS) using IQ-TREE and the differences in gene-wise log-likelihood scores (ΔGLS) between conflicting topologies using the Perl scripts of Shen et al. (2021). These analyses allowed us to quantify the phylogenetic signal distribution of alternative topologies at the site and gene levels, and to visualize the proportion of genes that support each topology. The three sets examined were as follows: (a) EJ for the sister relationship of Poaceae, EJ1 of [Poaceae, (Ecdeiocoleaceae, Joinvilleaceae)] vs. EJ2 of [Joinvilleaceae, (Ecdeiocoleaceae, Poaceae)]; (b) BT for the relationship of the early diverging group of Poales, BT1 of [(Typhaceae, Bromeliaceae), (Rapateaceae, core Poales)] vs. BT2 of Typhaceae, [Bromeliaceae, (Rapateaceae, core Poales)] vs. BT3 of Bromeliaceae [Typhaceae, (Rapateaceae, core Poales)]; and (c) EMX for the relationship of Mayacaceae, Eriocaulaceae, and Xyridaceae, EMX1 of EMX1 of Mayacaceae [Eriocaulaceae, (Xyridaceae (restiids, graminids))] vs. EMX2 of (Mayacaceae, Eriocaulaceae), [Cyperids, (Xyridaceae (restiids, graminids))] vs. EMX3 of (Mayacaceae, Eriocaulaceae), [(restiids, graminids), Xyridaceae].

In the analysis of supermatrix, just one or two outlier genes could have a significant effect on the phylogenetic topology (Brown and Thomson, 2016; Shen et al., 2017; Walker et al., 2019; Yang et al., 2021). In order to test this potential effect, we re-built phylogenies after removing the outlier genes in these matrices. The outlier genes were defined as those with phylogenetic signals deviating from a Gaussian-like distribution (Zhang R. et al., 2020; Yang et al., 2021). After removing these genes (Supplementary Table 5), we obtained 12 matrices (Table 1), i.e., 80PG_outlier_removed_EJ12 (*ycf*2 removed), 80PG-all_outlier_removed_EJ12 (*ycf*2), 80PG-half_outlier_ removed_EJ12 (*ycf*2), 80PG-no_outlier_removed_EJ12 (*ndh*F), 80PG_outlier_removed_BT123 (*ycf*3 and *pet*D), 80PG-all_outlier_removed_BT123 (*ycf*3 and *pet*D), 80PG-half_outlier_removed_BT123 (*ycf*3 and *pet*D), 80PG-no_outlier_removed_BT123 (*ycf*3), 80PG_outlier_removed_EMX123 (*ycf*1 and *ycf*2), 80PG-all_outlier_removed_EMX123 (*ycf*1 and *ycf*2), 80PG-half_outlier_removed_EMX123 (*ycf*1), and 80PG-no_outlier_removed_EMX123 (*ndh*A and *psa*B), for analyses.

### Test of Topological Concordance

We further tested the conflict and the concordance of gene trees and species trees of 80PG and 80PG-no matrices using PhyParts (Smith et al., 2015). We used the Phyx v1.01 (Brown, 2019) to re-root the gene tree and species tree. Meanwhile, the BS support value that is higher than 50% was retained while those lower than 50% did not provide conflict or concordance information. We then separately mapped the 80 and 72 gene trees onto the corresponding species trees by PhyPart. The output of PhyPart was visualized by the script, phypartspiecharts.py (Johnson, 2020).

## Results

### Taxon Sampling, Plastome, and Mitochondrial Matrix Characteristics

We newly assembled plastomes for 59 species and obtained 53 complete plastomes and 11 plastomes with gaps. The majority of these genomes were annotated to have 68–80 protein-coding genes, 4 rRNA genes, and 16–30 tRNA genes. The two main plastid gene matrices included 114 genes (114PG) and 80 genes (80PG), and the aligned sequences were 83,266 bp and 75,593 bp, respectively. The proportion of missing data ranged from 0.00 to 13.8% for the 11 matrices.

Three complete mitochondrial genomes and 46 at the scaffold level were extracted for 11–28 protein-coding genes for phylogenetic analysis. The alignment of the 28MG matrix was 23,709 bp in length. The proportion of missing data of four matrices ranged from 24.90 to 26.40%. The detailed information of all 15 matrices can be found in Table 1.

### Plastid Phylogenetic Tree

**FIGURE 1:**
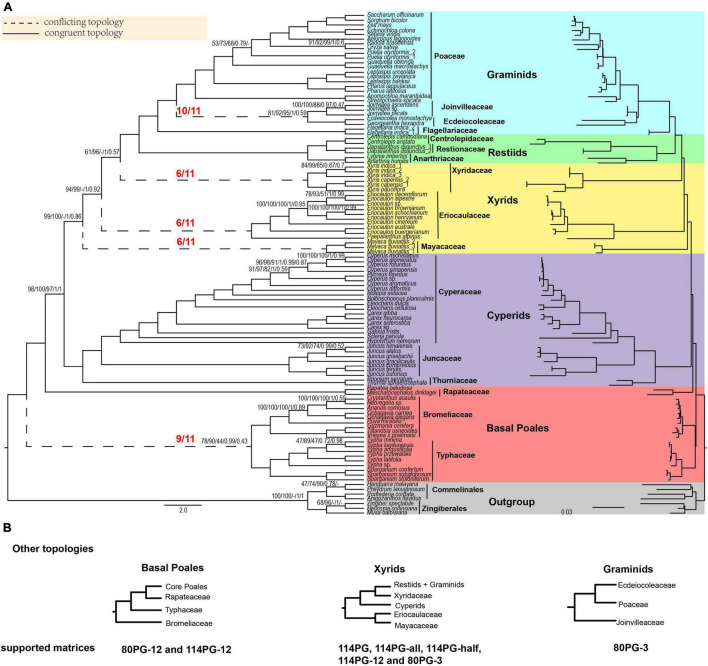
Poales phylogeny based on plastid genes and main conflicting topologies. **(A)** The Poales phylogeny is based on 80 plastid genes using Gblocks with no gap positions allowed (80PG-no). The red fractions on the branches represented by dotted lines indicate the supported numbers in the 11 matrices. Support values are given above branches with the order RAxML bootstrap (MLBS) values/IQ-TREE bootstrap (UFBS) values/MP bootstrap (MPBS)/posterior probability (PP) values/local posterior probability (LPP) values. Support values are only displayed for branches with BS support less than 100%, posterior probability less than 1.00, or local posterior probability less than 1.00. The conflicting topologies are indicated by “–”. Different colors represent different clades: Early diverging Poales in red, cyperids in purple, xyrids in yellow, restiids in green, graminids in blue, and the outgroup in gray. **(B)** Other topologies are different from panel **(A)** of basal Poales, xyrids, and graminids recovered in corresponding matrices. Note: -all, -half, and -no mean that using Gblocks with allowing gap positions with all, with half, and none. -12 mean 1st + 2nd condon position of the matrix; -3 mean 3rd codon position of the matrix.

For ML analyses, phylogenetic trees of Poales constructed using 11 plastid matrices shared a largely consistent topology, except that the relationships among the three families of Mayacaceae, Eriocaulaceae, and Xyridaceae were different (Figure 1 and Supplementary Figures 1–10). The support values of different matrices varied narrowly with generally high support (Figure 1 and Supplementary Figures 1–10). Therefore, the ML topology of the 80 PG-no matrix was selected for discussion, and the BS of RAxML (MLBS) and IQ-TREE (UFBS) were provided on branches.

In the phylogenetic trees of nine plastid matrices, the grouping of Typhaceae and Bromeliaceae was the first lineage diverging within the Poales (Figure 1 and Supplementary Figures 1–10). This node received weak to strong support (BS: 58–90%), and then sister to Rapateaceae and the grouping of the other families with full support. For the remaining two matrices of the 1st + 2nd codon positions of 114PG-12 and 80PG-12, the Bromeliaceae was the earliest diverging lineage with weak support (BS 48–70%).

The cyperid clade received full support in all 11 matrices. In addition, the phylogenetic relationships within it were identical in all the matrices with the Cyperaceae sister to Juncaceae and then the sister to Thurniaceae (Figure 1 and Supplementary Figures 1–10).

There were two different topologies found for the xyrid clade (Figure 1 and Supplementary Figures 1–10). For the first one, the Eriocaulaceae was the sister to Mayacaceae with a close relationship to the cyperid clade with weak to strong support (BS = 58–99%), and the Xyridaceae became an independent lineage that was close to the restiid clade in five matrices (114PG, 114PG-all, 114PG-half, 114PG-12, and 80PG-3) with full support. For the second one, the xyrid clade was collapsed with Mayacaceae, Eriocaulaceae, and Xyridaceae diverging sequentially along the backbone phylogeny of Poales and the support was BS = 72–99, 61–100, and 100%, respectively.

In the 11 matrices, the restiid clade was all fully supported. The Restionaceae was sister to Centrolepidaceae, and then sister to Anarthriaceae with full support (Figure 1 and Supplementary Figures 1–10). The restiid clade was sister to the graminid clade, which was fully supported in all the 11 matrices. The Flagellariaceae was sister to the other three families in all matrices with full support. The Ecdeiocoleaceae was sister to Joinvilleaceae in 10 matrices with weak to strong support (BS = 57–92%) and then sister to Poaceae with full support. The 80PG-3 matrix found the grouping of Ecdeiocoleaceae and Poaceae with moderate support (BS = 70–87%) and then sister to Joinvilleaceae with full support (Figure 1 and Supplementary Figures 1–10).

We selected two matrices (80PG and 80PG-no) to run ASTRAL, MP, and BI analyses. For ASTRAL, the early diverging topology of [(Bromeliaceae, Typhaceae) (Rapateacaea, core Poales)] received weak support in 80PG (LPP = 0.61) and 80PG-no (LPP = 0.43). The sister group of Ecdeiocoleaceae and Joinvilleaceae was found in 80PG-no with weak support (LPP = 0.59), while Joinvilleaceae diverged first followed by Ecdeiocoleaceae + Poaceae with moderate support (LPP = 0.86) in 80PG. The other relationships were similar to 80PG-no by ML analyses (Figure 1 and Supplementary Figures 11, 12).

For MP analyses, the early diverging group of 80PG and 80PG-no was Bromeliaceae + Typhaceae with weak support of maximum parsimony BS (MPBS) = 58 and 44%, respectively, and Ecdeiocoleaceae + Joinvilleaceae was sister to Poaceae with moderate to strong support (MPBS = 74 and 94%). The 80PG matrix found that Eriocaulaceae was sister to Xyridaceae with weak support (MPBS = 53%) and then sister to Mayacaceae with weak support (MPBS = 54%). In the 80PG-no matrix, we obtained a clade of Eriocaulaceae + Mayacaceae with weak support (MPBS = 51%) which was sister to Xyridaceae with strong support (MPBS = 98%). The topology of the other clades was similar to the ML analyses (Figure 1 and Supplementary Figures 13, 14).

For BI analyses, 80PG and 80PG-no generated the same topology as the ML analyses (114PG-no, 80PG, 80PG-all, 80PG-half, and 80PG-no). The Bromeliaceae + Typhaceae clade was the early diverging group with strong support [posterior probability (PP) = 0.99] in two matrices. The Ecdeiocoleaceae + Joinvilleaceae clade was sister to Poaceae with full support (PP = 1.00) in 80PG-no and weak support (PP = 0.52) in 80PG. The internal relationship of the xyrid clade was Eriocaulaceae, Mayacaceae, and Xyridaceae diverging in sequence. The topology of the other branches was similar to the ML analyses (Figure 1 and Supplementary Figures 15, 16).

### Mitochondrial Phylogenetic Tree

**FIGURE 2:**
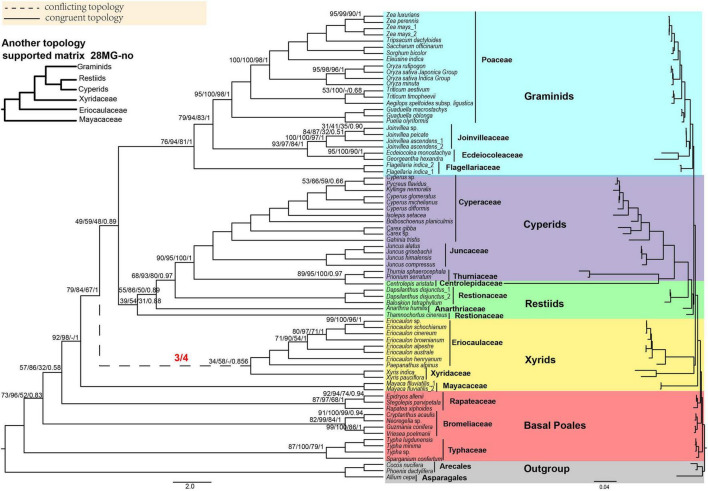
The Poales phylogeny based on 28 mitochondrial genes (28MG). The red fractions on the branches represented by dotted lines indicate the supported numbers in the four matrices. Support values are given above branches with the order MLBS values/UFBS values/MPBS/PP values/LPP values. Support values are displayed for branches with less than 100% BS support, less than 1.00 for posterior probability, or less than 1.00 for local posterior probability. The conflicting topologies are shown by “–”. The clades are shown in different colors in Figure 1. The top left corner shows another topology that is different from the 28 MG matrix of xyrids recovered in the corresponding matrix. Note: -no mean that using Gblocks that allow gap positions with none.

We used four matrices of mitochondrial genes to reconstruct the phylogenetic tree of Poales. In ML analyses, the topologies remained the same except for the phylogenetic relationships involving the three families of Eriocaulaceae, Mayacaceae, and Xyridaceae. In all four matrices, the early diverging group was Typhaceae followed by Bromeliaceae with weak to strong support BS = 72–99% (Figure 2 and Supplementary Figures 17–22). The xyrid clade was revealed to be paraphyletic. In the IQ-tree, the Mayacaceae diverged first and was followed by Eriocaulaceae + Xyridaceae in the 28MG and 28MG-all matrices, and the support for these two nodes was weak BS (38–69%) (Figure 2 and Supplementary Figure 18). For the 28MG-half and 28MG-no matrices, the Mayacaceae also diverged first while the relationship between Eriocaulaceae and Xyridaceae was unresolved (Supplementary Figures 20, 22). In RAxML, the Mayacaceae diverged first, and Eriocaulaceae and Xyridaceae successively diverged in only 28MG-no matrix with weak support (MLBS = 26–71%) (Supplementary Figure 21) and they were sisters to each other with weak support of MLBS = 34–42% for the other three matrices. The restiid clade also became paraphyletic and the Anarthriaceae (*Anarthria humilis* Nees) was embedded in the Restionaceae with weak to strong support BS = 68–93%. The Restionaceae was sister to the cyperid clade with strong support (BS = 85–97%). Surprisingly, the Centrolepidaceae was also embedded in the Cyperid clade and was sister to Thurniaceae with strong support (BS = 85–97%). The sister relationship between Thurniaceae + Centrolepidaceae and Cyperaceae + Juncaceae received full support. The topology of the graminid clade in all the matrices is consistent with the Flagellariaceae in the basal position and Ecdeiocoleaceae + Joinvilleaceae sister to Poaceae with strong support (BS = 89–100%) except for the 28MG-no matrix with weak support (MLBS = 60%) in RAxML (Figure 2 and Supplementary Figures 17–22).

For MP analyses, we only analyzed the 28MG matrix. The early diverging family was the Typhaceae and then Bromeliaceae followed by Rapateaceae with MPBS of 52, 32, and 92% (Supplementary Figure 23), respectively. Afterward, the Mayacaceae was sister to Xyridaceae with weak support (MPBS = 68%) and the Eriocaulaceae formed a single clade. The Ecdeiocoleaceae and Joinvilleaceae were sisters with moderate support (MPBS = 84%) and as sisters to Poaceae with strong support (MPBS = 98%). The topologies of other branches were concordant with the RAxML tree of 28MG (Figure 2 and Supplementary Figure 23).

For BI analyses, the topology of 28MG was consistent with the RAxML tree of this matrix (Figure 2 and Supplementary Figure 24). The early diverging families of Typhaceae, Bromeliaceae, and Rapateaceae were separated in turn with PP = 0.83, 0.58, and 1.00. The Ecdeiocoleaceae was sister to Joinvilleaceae and this grouping was then sister to Poaceae and also with full support (Supplementary Figure 24).

### Quantification of Branch Support Values

We chose two matrices (80PG-no and 28MG) to quantify branch support values. The QC score of ≥0.5 was considered to be strong to manifest support among quartets (Pease et al., 2018; Larson et al., 2020). The full support (QC = 1) was obtained for the monophyly of each family (Supplementary Figure 25).

In the 80PG-no matrix, we found no support (QC = -0.048) for Bromeliaceae + Typhaceae (Supplementary Figure 25). The monophyly of cyperid and restiid was in full support (QC = 1). The sister relationship between Thurniaceae and Cyperaceae + Juncaceae was also in full support (QC = 1). The Mayacaceae, Eriocaulaceae, and Xyridaceae diverged in sequence with no support (QC = -0.34 and -0.2) and weak support (QC = 0.12) for the node placing Xyridaceae sister to restiid + graminid. Within the graminid clade, the Ecdeiocoleaceae + Joinvilleaceae received moderate support (QC = 0.38) while receiving full support (QC = 1) for this grouping as sister to Poaceae. In contrast, the QD = 0 denoted strong alternative relationships about the nodes within the graminid clade, and in general, the scores of less than 0.3 could be considered as that discordant quartets tend to be heavily skewed toward the conflicting topology (Pease et al., 2018; Larson et al., 2020). The sister groups of Ecdeiocoleaceae and Joinvilleaceae had a low QD score of 0.017 indicating the skew in discordance meaning the possible presence of a supported secondary evolutionary history. Similarly, the nodes connecting Mayacaceae, Eriocaulaceae, and Xyridaceae also received low QD scores (0.27 and 0.044) and thus strong support for alternative evolutionary history. The QD score for the Bromeliaceae + Typhaceae was 0.61, which indicated that inconsistent topologies occurred with relatively equal frequency. The QI scores for all the nodes and the relationships between families were all above 0.76, meaning enough phylogenetic information for these nodes.

For the mitochondrial matrix 28MG, we also obtained no-support (QC = −0.039) for the node placing the Bromeliaceae as the first diverging lineage within the Poales, as well as for the Eriocaulaceae + Xyridaceae (QC = −0.63) (Supplementary Figure 26). Moreover, the support was weak for the phylogenetic relationships within the graminid clade with QC scores from 0.13 to 0.37. The QD scores for the nodes connecting Bromeliaceae and Typhaceae and Eriocaulaceae + Xyridaceae were the same as 0.16, indicating a majority of quartets supporting one of the alternative discordant quartet arrangements. The sister relationship between Cyperaceae + Juncaceae and Thurniaceae + Centrolepidaceae had QD = 0 and all the discordant trees sampled were only one of the two alternative topologies. The QI score for the node leading to Typhaceae and Rapateaceae was 0.4, while the other interfamilial relationships were all supported by QI > 0.68.

### Quantification of Phylogenetic Signals for Alternative Topologies

**FIGURE 3:**
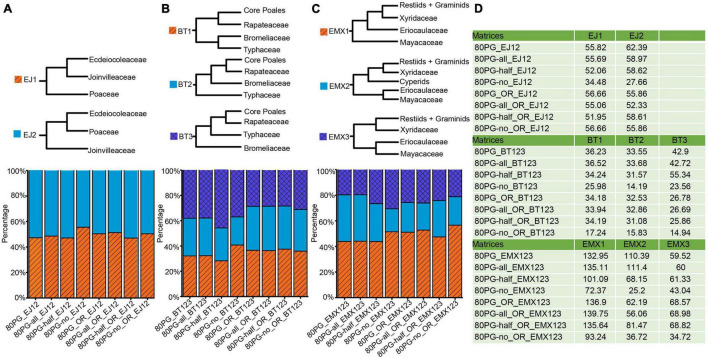
Proportions of phylogenetic signals (ΔGLS) supporting alternative topologies of three conflicting nodes for each of 8 data matrices. **(A)** Proportions of ΔGLS supporting either of two alternative relationships among Ecdeiocoleaceae, Joinvilleaceae, and Poaceae family across eight matrices; **(B)** Proportions of ΔGLS supporting either of the three alternative relationships among Bromeliaceae, Rapateaceae, Typhaceae, and the rest across eight matrices; **(C)** Proportions of ΔGLS supporting either of the three alternative relationships among Eriocaulaceae, Mayacaceae, Xyridaceae, cyperids and the rest across eight matrices; **(D)** the summed ΔGLS values for each matrix. Note: -all, -half, and -no mean that using Gblocks allow gap positions with all, with half, and none. The value −12 means 1st + 2nd condon positions of the matrix; The value −3 means the 3rd codon position of the matrix. The notations _OR and _outlier_removed mean removing outlier genes.

We examined phylogenetic signals for three major conflicting topologies for the Poales phylogeny. Phylogenetic signals for the alternative resolutions of each conflicting topology are shown in Figure 3 and Supplementary Table 4. For the conflict involving Ecdeiocoleaceae and Joinvilleaceae, we examined ΔGLS values between EJ1 and EJ2. The proportions of phylogenetical signals for EJ1 and EJ2 were basically the same (EJ1: 47.04–55.49% vs EJ2: 44.51–52.96%). We found nearly identical proportions of signals of 46.99–51.27% and 49.64–53.01% in the four new matrices for EJ1 and EJ2 after removing the outlier genes (Supplementary Table 5), respectively.

For the Bromeliaceae and Typhaceae, computation of ΔGLS values showed ambiguous proportions of sites supporting anyone of the three different topologies of BT1, BT2, and BT3 (Figure 3). The proportions of phylogenetical signal for BT1, BT2, and BT3 ranged from 28.26 to 40.77%, from 22.27 to 29.83%, and from 36.97 to 45.68%, respectively. The 80PG-half showed a higher proportion of phylogenetic signals supporting BT3 (45.68%). After removing the outlier genes, we found that the proportions of signals in the four new matrices of BT1, BT2, and BT3 were still low from 35.91 to 37.52%, from 32.97 to 35.15%, and from 28.38 to 31.12%, respectively.

For the relationships among the three families of Eriocaulaceae, Mayacaceae, and Xyridaceae, computation of ΔGLS values also showed ambiguous proportions of sites (Figure 3). The higher proportions of phylogenetic signal from 43.84 to 51.47% were shown for EMX1 while lower values from 17.92 to 36.45% and from 19.58 to 30.61% were obtained for EMX2 and EMX3, respectively. We observed increased proportions of signals for EMX1 while decreased values for EMX 2 and EMX3 after removing the outlier genes.

### Test of Topological Concordance

The topology of ASTRAL trees of the 80PG and 80PG-no matrix was a bit different (Supplementary Figure 27). The one was about the relationship among Eriocaulaceae, Mayacaceae, and Xyridaceae, and another involved Ecdeiocoleaceae and Joinvilleaceae. In 80PG, the Mayacaceae diverged first and Eriocaulaceae and Xyridaceae formed a sister group, while the three families diverged sequentially in 80PG-no. The other was about whether the Joinvilleaceae diverged first (80PG) or grouped with the Ecdeiocoleaceae (80PG-no).

Within the 80 genes of the 80PG matrix, only two genes supported the Bromeliaceae + Typhaceae, but 10 genes against this topology and the remaining genes were uninformative on these relationships. In comparison, none supported and seven genes rejected this topology out of the 72 genes in the 80PG-no matrix. The cyperid clade had 41 genes supported and 15 genes rejected from the 80 genes and a high number of 51 genes supported from the 72 genes, indicating that this clade is stable.

The two nodes connecting Eriocaulaceae, Mayacaceae, and Xyridaceae conflicted with a few supported genes of three and four, respectively, and both had 17 rejected genes in 80PG. In 80PG-no, the Eriocaulaceae + Xyridaceae also only had seven supported genes but 23 rejected genes. In contrast, the restiid clade was stable with high support of 38 and 52 genes in 80PG and 80PG-no, respectively. There were more rejected genes than supported genes for the Ecdeiocoleaceae + Joinvilleaceae (20 vs. 12) in 80PG-no and the first divergence of the Joinvilleaceae (26 vs. 14) in 80PG.

### Comparison of Organelle and Nuclear Phylogenies

**FIGURE 4:**
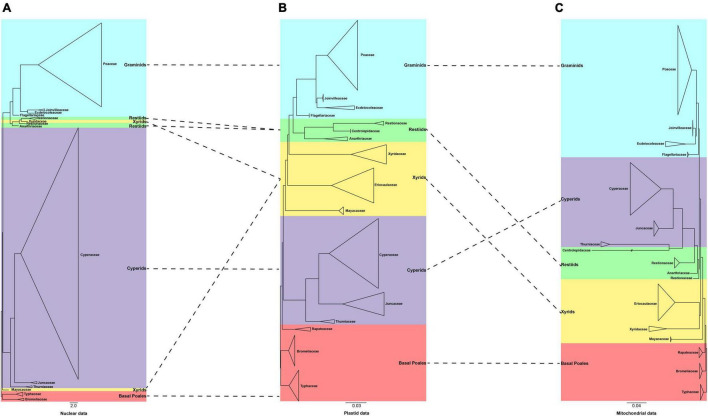
A comparison of the phylogenies obtained from the plastid, mitochondrial, and nuclear matrices. **(A)** 353 nuclear data set [adopted from Baker et al. (2021)] **(B)** 80 plastid data set **(C)** 28 mitochondrial data set. The clades are shown in different colors in Figure 1. The branch with “//” was shortened by 50%.

We further compared our plastid and mitochondrial phylogenies with the recently published nuclear tree of Poales (Baker et al., 2021; Figure 4). We found that the position of the graminid remained unchanged. The conflict concentrated on the early diverging group, the cyperid and restiid clades, and the xyrid assembly. Taking the plastid tree as a reference, we explored the conflict in detail. In the plastid phylogeny, the phylogenetic placement of the families of the xyrid assembly is between the cyperid clade and the restiid clade, while in the mitochondrial phylogeny, the position changes and is located between the early diverging taxa and the cyperid clade. For the nuclear data, the placement of the Mayacaceae is the same as that of mitochondria and the Xyridaceae is clustered into the Restionaceae (Baker et al., 2021). The cyperid clade has a close relationship with the early diverging group in the plastid data, while it is located between the xyrid assembly, Mayacaceae and restiid clade in the nuclear data (Baker et al., 2021), and between the graminid clade and the restiid clade in the mitochondrial data. The restiid clade is sister to the graminid clade in the plastid and nuclear data while sister to the cyperid clade in the mitochondrial data.

## Discussion

### The Early Diverging Poales

The early diverging Poales include three families: Bromeliaceae, Rapateaceae, and Typhaceae. However, the relationships among them are variable in recent studies. The first divergence of Bromeliaceae was supported by the analyses of 75, 77, or 81 plastid genes (Givnish et al., 2010, 2018; Barrett et al., 2016) and the combination of one mitochondrial gene, two rDNA genes, and four plastid genes (Chase et al., 2006). In contrast, Typhaceae was estimated as the first diverging lineage followed by Bromeliaceae based on the analyses of 81 plastid trees in other studies (Darshetkar et al., 2019; Li et al., 2019) or nuclear genes (McKain et al., 2016). Moreover, the sister relationship between Bromeliaceae and Typhaceae was also revealed in some studies, such as using the *rbc*L and *ndh*F genes (Christin et al., 2008; Bouchenak-Khelladi et al., 2014), the low-copy nuclear gene *PHYC* (Hertweck et al., 2015), and 353 nuclear genes (Baker et al., 2021). Analyses of the concatenated plastid genes all uncovered the sister relationship of Bromeliaceae and Typhaceae with weak to strong support (Figure 1 and Supplementary Figures 1–10). Similarly, the coalescent ASTRAL analyses also supported Bromeliaceae as sister to Typhaceae despite weak support (Supplementary Figures 11, 12).

Nevertheless, the mitochondrial trees revealed the Typhaceae as the earliest diverging group with weak to strong support (Figure 2 and Supplementary Figures 17–24), and this result is in conflict with the plastid phylogeny. The conflict between plastid and mitochondrial phylogenies may be due to the evolutionary histories of these two subcellular compartments being unlinked (Sun et al., 2015) and/or incomplete lineage sorting (Lee et al., 2018). The branches leading to the Bromeliaceae and Typhaceae are very short, indicating that they may endure extreme changes in the rates of molecular evolution (Givnish et al., 2018). Moreover, the short internal branches and conflicting topology of Typhaceae and Bromeliaceae in trees could also be due to rapid radiation as suggested before (hence lack of sufficient phylogenetic signals) (Barrett et al., 2013; Hertweck et al., 2015; Hochbach et al., 2018). Taken together, we consider the phylogenetic relationship between Bromeliaceae and Typhaceae as polytomy Hochbach et al. (2018) and as the earliest diverging lineages of Poales followed by Rapateaceae.

### The Non-monophyly of the Xyrid Assembly

The phylogenetic relationships among Eriocaulaceae, Mayacaceae, and Xyridaceae within the xyrid clade remain unresolved in previous studies. The Eriocaulaceae was placed as sister to Xyridaceae (Christin et al., 2008) or Mayacaceae (Givnish et al., 2010) in analyses using plastid genes, while the recent plastome study recognized the topology of [Mayacaceae, (Eriocaulaceae, (Xyridaceae, (restiids, graminids)))] (Li et al., 2019). Using 353 nuclear genes, Baker et al. (2021) suggested that the Mayacaceae was an early diverging lineage within the Poales following the divergence of Bromeliaceae and Typhaceae, while the Xyridaceae was embedded in the restiid clade.

Five of our concatenated plastid datasets (114PG, 114PG-all, 114PG-half, 114PG-12, and 80PG-3) revealed Eriocaulaceae as sister to Mayacaceae with a close relationship to the cyperid clade, and the Xyridaceae became an independent clade that was close to the restiid clade (Supplementary Figures 1–3, 5, and 10). The other six matrices supported the topology [Mayacaceae, (Eriocaulaceae, (Xyridaceae, (restiids, graminids)))] (Figure 1 and Supplementary Figures 4, 6–9). The two conflicting topologies are obtained probably, as a result, the tRNA and rRNA genes have different evolutionary histories from the protein-coding genes in the plastid genome, e.g., faster substitution rate. The third codon positions can mutate more frequently than the first and second positions and thus may experience mutation saturation leading to the phylogenetic artifact (Zeng et al., 2017). In comparison, the ASTRAL analyses revealed the topology [Mayacaceae, ((Eriocaulaceae, Xyridaceae), (restiids, graminids))] and [Mayacaceae, (Eriocaulaceae, (Xyridaceae, (restiids, graminids)))] with weak and strong support in 80PG and 80PG-no, respectively (Supplementary Figures 11, 12). For the mitochondrial data, two different topologies emerged, [Mayacaceae, (Eriocaulaceae, (Xyridaceae, (restiids, graminids)))] and [Mayacaceae, ((Eriocaulaceae, Xyridaceae), (restiids, graminids))]. This result is likely due to the insufficient informative sites in the mitochondrial genes. Whatever be the final phylogenetic resolution of the xyrid clade, it appears that the monophyly of this clade would not be achieved and we can consider it paraphyletic. However, for the convenience of communication, we suggest tentatively keeping the name and calling it the paraphyletic xyrid assembly.

### The Restiid Clade

The internal relationship of the restiid clade is still doubtful (Briggs et al., 2014; Hochbach et al., 2018). Previous studies based on the plastid, nuclear, or combined plastid, nuclear, and mitochondrial data (Chase et al., 2006; Bouchenak-Khelladi et al., 2014; Hochbach et al., 2018; Baker et al., 2021) supported a monophyletic Anarthriaceae, which was sister to the Restionaceae. However, the relationship between Centrolepidaceae and Restionaceae had two different topologies. The sister relationship between Centrolepidaceae and Restionaceae was supported by a majority of previous studies with plastid, nuclear, and mitochondrial data (e.g., Chase et al., 2006; Givnish et al., 2010; Bouchenak-Khelladi et al., 2014; McKain et al., 2016; Darshetkar et al., 2019; Li et al., 2019; Baker et al., 2021). In other studies, the Centrolepidaceae was embedded in Restionaceae based on *rbc*L and *atp*B (Bremer, 2002) and the combined data (*mat*K, *Phy*B, and *Topo*6) (Hochbach et al., 2018). In our study, the topology of [Anarthriaceae, (Restionaceae, Centrolepidaceae)] is revealed by plastid data with strong support in both the concatenate and coalescent methods.

However, in our mitochondrial data, the Anarthriaceae is embedded in the Restionaceae. This result may be due to the fact that only one mitochondrial gene of *cob* was available for *Thamnochortus cinereus* H.P. Linder in analyses and this gene is short of phylogenetic information. Moreover, the Centrolepidaceae is placed in the cyperid clade and as a sister to the Thurniaceae with strong support. The delimitation of the Restionaceae and the placement of the Centrolepidaceae has thus not been fully resolved, and more molecular data and taxon samplings are needed for further analysis.

### The Graminid Clade

The monophyly of the graminid clade with the Flagellariaceae as the first diverging group is supported by all previous studies (e.g., Chase et al., 2006; Christin et al., 2008; Soltis et al., 2011; Bouchenak-Khelladi et al., 2014; Hertweck et al., 2015; McKain et al., 2016; Hochbach et al., 2018; Darshetkar et al., 2019; Li et al., 2019; Baker et al., 2021). However, the relationship within the remaining families of Ecdeiocoleaceae, Joinvilleaceae, and Poaceae is still blurry. Both the topologies of [Joinvilleaceae, (Ecdeiocoleaceae, Poaceae)] and [(Joinvilleaceae, Ecdeiocoleaceae), Poaceae] were revealed in previous studies (Givnish et al., 2010; Bouchenak-Khelladi et al., 2014; Barrett et al., 2016; McKain et al., 2016; Hochbach et al., 2018; Darshetkar et al., 2019; Li et al., 2019; Baker et al., 2021). Our study supports the latter topology using the concatenate method, as well as the coalescent analysis of the 80PG. In contrast, the coalescent analysis of the 80PG-no generates the topology [Joinvilleaceae, (Ecdeiocoleaceae, Poaceae)] with moderate support. The conflict between coalescent and concatenate methods could be caused by the limitations of ASTRAL when largely uninformative loci exist as in the 80PG-no (Yang et al., 2021). The mitochondrial data also support the topology of [(Joinvilleaceae, Ecdeiocoleaceae), Poaceae] with high support values and we advocate a topology of [Flagellariaceae, ((Joinvilleaceae, Ecdeiocoleaceae), Poaceae)] for the graminid clade.

### Conflicting Signals in Plastid Phylogeny

Based on branch support value and phylogenetic signal analyses, we observed the extensive presence of conflicts among plastid loci involving several long-questioned nodes (e.g., the relationship of Bromeliaceae and Typhaceae, Joinvilleaceae and Ecdeiocoleaceae, and the xyrid assembly). The no-support means a majority of quartets favor one of the alternative discordant quartet arrangement history (Pease et al., 2018). We found no support for the topologies [(Bromeliaceae, Typhaceae), (Rapateaceae, core Poales)] and [Mayacaceae, (Eriocaulaceae, (Xyridaceae, (graminids, restiids)))]. The ΔGLS values show almost the same proportions of sites supporting three kinds of topologies referring to the Bromeliaceae and Typhaceae, illustrating the conflict with three different topologies, and their relationships may be better treated as polytomy at present. In addition, the topology of [Mayacaceae, (Eriocaulaceae, (Xyridaceae, (graminids, restiids)))] and [(Joinvilleaceae, Ecdeiocoleaceae), Poaceae] both have low QD scores, suggesting that there is a skew between two inconsistent topologies. This result points to a given branch having a biased biological process other than the background lineage sorting, including confounding variables, such as introgression, high heterogeneity of evolutionary rate, heterogeneous base composition, etc. (Pease et al., 2018).

The ΔGLS values show the higher proportions of phylogenetic signal for [Mayacaceae, (Eriocaulaceae, (Xyridaceae, (graminids, restiids)))], and the maximum is 47.5%, but the other two topologies have about 50%. Previous studies have suggested that removing problematic sequences would avoid artifacts to some extent and contribute to the robustness of phylogenetic results (Goremykin et al., 2010; Parks et al., 2012; Yang et al., 2021), although identifying these sequences is difficult (Som, 2015). We removed the outlier genes but found that they have little effect on resolving these long-debated relationships between Mayacaceae, Eriocaulaceae, and Xyridaceae. Recently, Doyle (2021) stressed that the plastid genome should be treated as a single unit for phylogenetic analyses. However, some studies indicate that it is not proper to treat the plastid genome as a single unit particularly when the evidence of recombination is present (Gonçalves et al., 2020; Daniell et al., 2021; Yang et al., 2021). Several studies have shown that the conflicts within the plastid genome (Walker et al., 2019; Zhang R. et al., 2020; Yang et al., 2021), and our results also support this point. However, the cause of the plastid conflict has not yet been determined. Stochastic errors, such as those associated with rapid radiation and limited phylogenetic signals may explain the majority of the observed conflicts (Zhang R. et al., 2020; Yang et al., 2021). In plastid phylogenetic analyses, the vast majority of plastid loci are generally uninformative, and a few genes with strong signals will largely determine the phylogenetic resolution as shown here (Supplementary Figure 27; Walker et al., 2019; Zhang R. et al., 2020; Yang et al., 2021). The biological cause for the conflict of plastids could be heterogeneous recombination and a gene transfer between genomes (Walker et al., 2019; Zhang R. et al., 2020; Daniell et al., 2021; Yang et al., 2021). However, we did not find any clues for these factors playing a role here. Instead, the phylogenetic tree of the Poales harbors numerous contrasting short and long branches, meaning highly heterogeneous plastome evolutionary rates among these families of the Poales, and this could be one of the reasons for causing conflicts within the plastid genomes (Barrett et al., 2013, Barrett et al., 2016). The species of Poales have a variety of habitats, and ten families of them grow in swamp or wet habitats, among which Typhaceae and Mayacaceae are typical aquatic plants (Linder and Rudall, 2005). The plastome of aquatic plants may have a more complex structure, and the deletion and inversion were found in a large number of aquatic plants, e.g., *Eleocharis* (Cyperaceae) and *Najas flexilis* (Willd.) Rostk. and W.L.E. Schmidt (Hydrocharitaceae) (Peredo et al., 2013; Lee et al., 2020). This may also contribute to the phylogenetic conflict. In addition, the photosynthetic pathways of Poales are also diverse, including all known three pathways of C_3_, C_4_, and Crassulacean acid metabolism (CAM) (Linder and Rudall, 2005). This is a potential explanation for the high heterogeneity of substitution rates heterogeneity, which ultimately results in phylogenetic conflict (Barrett et al., 2016).

### Conflicts Among Three Plant Genomes

The phylogenetic conflict between organelles (plastid and mitochondrial) and nuclear genes has been reported in various taxa, such as coralline red algae, *Lachemilla* (Rosaceae), and *Pterocarya* (Juglandaceae) (Lee et al., 2018; Morales-Briones et al., 2018; Mu et al., 2020). The plastid and mitochondrial genomes have a strong conflict in our study. The mitochondrial data have more missing data and lower coverage than the plastid data, which may be one of the factors causing the conflict between mitochondrial and plastid phylogenies. Another possible explanation is that the mitochondrial genomes of Poales have undergone extensive horizontal gene transfer between nuclear and plastid genomes, which is typical in land plants (Folk et al., 2016). In our study, we found that the systematic position of Centrolepidaceae is extraordinary and the branches are very long (Figure 2 and Supplementary Figures 19–23). This phenomenon is also observed by Folk et al. (2016), who indicates that the foreign DNA of the nucleus may be the cause for the large difference in branch length and location in mitochondrial analysis.

Meanwhile, strong conflicts are also detected between the organelle genomes and nuclear genes. The phylogenetic relationships of the three clades/assembly (cyperid, restiid, and xyrid) are different among the three genomes. This may be caused by insufficient sampling of nuclear data. Three families of Centrolepidaceae, Rapateaceae, and Eriocaulaceae are not sampled in our nuclear data, and the sampling distribution is uneven between each family. For example, the Xyridaceae have only one species included, which may be the reason for the odd phylogenetic position of this family. There is also a potential biological source of the incongruence among plastid, mitochondrial, and nuclear loci (Sun et al., 2015). The factors could be incomplete lineage sorting, hybridization, lateral transfer of organellar genomes, plastome capture, and polyploidy (Stegemann et al., 2012; Lee et al., 2018; Morales-Briones et al., 2018).

In particular, polyploidy is prevalent in plant groups and all the families of Poales have experienced polyploidization events in their evolutionary history (McKain et al., 2016; Van de Peer et al., 2017; Morales-Briones et al., 2018; Wu et al., 2020; Guo et al., 2021). Here, the sampled taxa of *Centrolepis aristata* (R.Br.) Roem. and Schult. (restiid) and the species of Eriocaulaceae (xyrid) are probably hexaploid (Šmarda et al., 2014), and *Juncus bufonius* L. (cyperid) is octoploid (Kubešová et al., 2010). Meanwhile, hybridization is ubiquitous in the green plant (Soltis and Soltis, 2009; Triplett et al., 2010), and the combination of hybridization and polyploid events (Soltis and Soltis, 2009) is another usual cause of phylogenetic conflict. The conflicts of three genomic data reflected in the branch lengths (Jost et al., 2021) indicate that the molecular evolution rate between different genomes is highly heterogeneous with certain families of the Poales experiencing an accelerated rate of sequence evolution (Guisinger et al., 2010; Barrett et al., 2013, Barrett et al., 2016). The fast-evolving sites are more likely to be saturated and prone to the accumulation of non-phylogenetic signals (Rodríguez-Ezpeleta et al., 2007), and thus leading to topological conflicts among the three genomes. In short, the conflict of three genomic data might be due to a combination of the missing data in mitochondria, polyploid history, heterogeneity of molecular evolution rate, and sparse sampling of nuclear data, and these factors deserve to be explored in detail in future studies. Furthermore, due to the limitations of the organelle genome, the nuclear genomic data will be used to finally resolve the phylogenetic relationships of Poales, so as to improve the understanding of the cause of phylogenetic conflict in this order.

## Conclusion

With a broad taxon sampling, we used plastid and mitochondria genomes to infer the phylogenetic tree of Poales and found the long-standing controversial nodes of Poales mainly caused by extensive conflict across genomic compartments. For the xyrid assembly, we found it paraphyletic, and its relationship with the three families, Eriocaulaceae, Mayacaceae, and Xyridaceae within the Poales is still not fully resolved. Our study has not only revealed phylogenetic conflicts within the plastid genomes, but also extensive conflicts among the plastid, mitochondrial and nuclear data in the Poales. Many factors, such as the missing data of mitochondrion, insufficient nuclear sampling, rapid radiation, heterogeneity of molecular evolution rate, and allopolyploidy by hybridization are potentially involved in generating these conflicts in the Poales.

## Data Availability Statement

The datasets presented in this study can be found in online repositories. The names of the repository/repositories and accession number(s) can be found in the article/Supplementary Material.

## Author Contributions

P-FM, D-ZL, and HW conceptualized the study. HW, P-FM, J-BY, and J-XL were involved in data generation and methodology. HW worked on data analysis and visualization. HW, P-FM, D-ZL, J-BY, and J-XL reviewed and revised the first draft of the manuscript and approved the submitted version.

## Conflict of Interest

The authors declare that the research was conducted in the absence of any commercial or financial relationships that could be construed as a potential conflict of interest.

## Publisher’s Note

All claims expressed in this article are solely those of the authors and do not necessarily represent those of their affiliated organizations, or those of the publisher, the editors and the reviewers. Any product that may be evaluated in this article, or claim that may be made by its manufacturer, is not guaranteed or endorsed by the publisher.

## Footnotes

1
https://github.com/ShuiyinLIU/QS_visualization
