# Supplementary material for: Organelle Phylogenomics and Extensive Conflicting Phylogenetic Signals in the Monocot Order Poales
Source: Front Plant Sci. 2022 Jan 31;12:824672. doi: 10.3389/fpls.2021.824672 (PMC8841755; doi:10.3389/fpls.2021.824672)
Supplement: Supplementary file 9 [file Data_Sheet_4.zip › Supplementary Figures/Supplementary Figure 26.pdf]

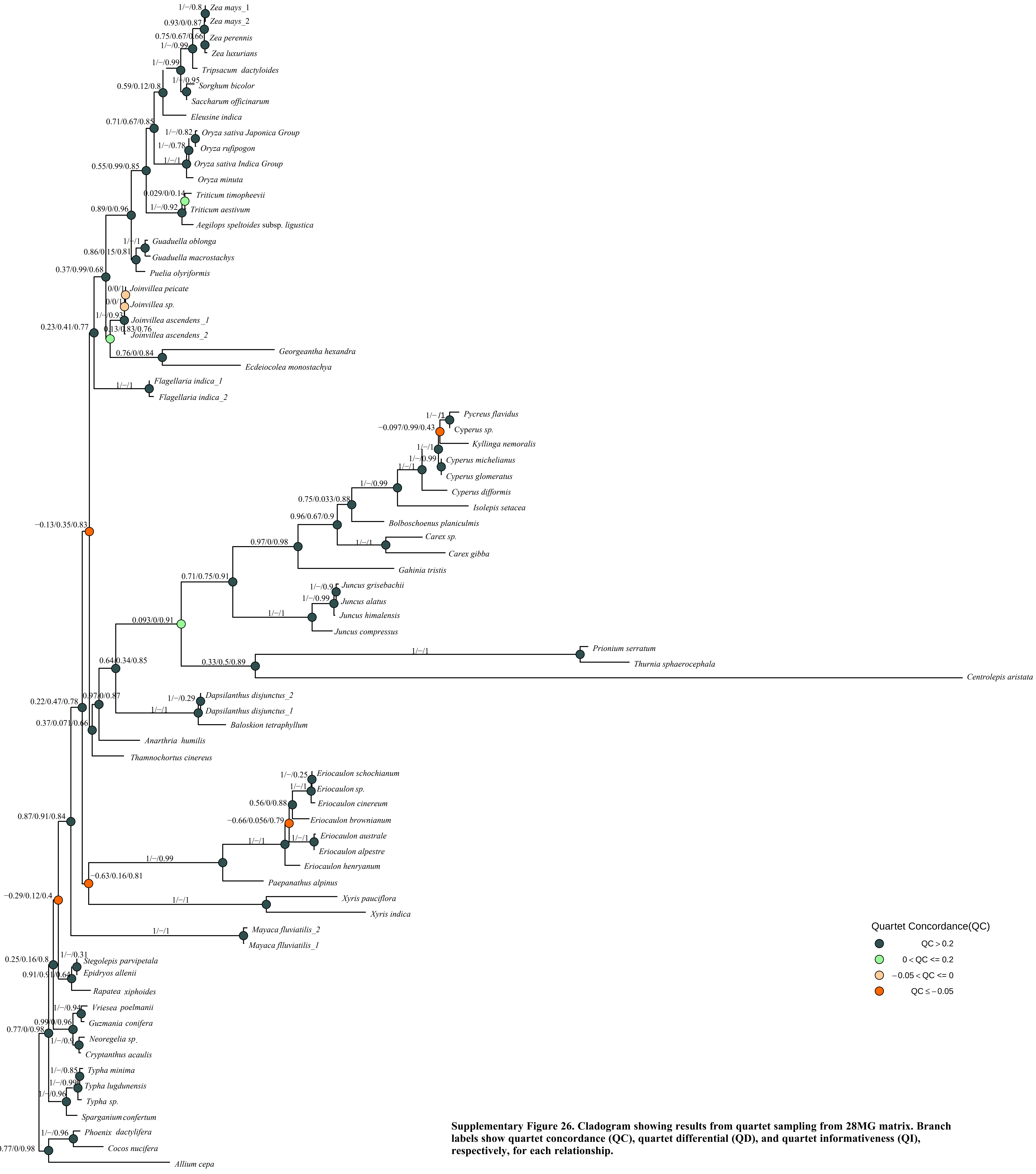

Supplementary Figure 26. Cladogram showing results from quartet sampling from 28MG matrix. Branch labels show quartet concordance (QC), quartet differential (QD), and quartet informativeness (QI), respectively, for each relationship.
