# Supplementary figures and images for: Organelle Phylogenomics and Extensive Conflicting Phylogenetic Signals in the Monocot Order Poales
Source: Front Plant Sci. 2022 Jan 31;12:824672. doi: 10.3389/fpls.2021.824672 (PMC8841755; doi:10.3389/fpls.2021.824672)

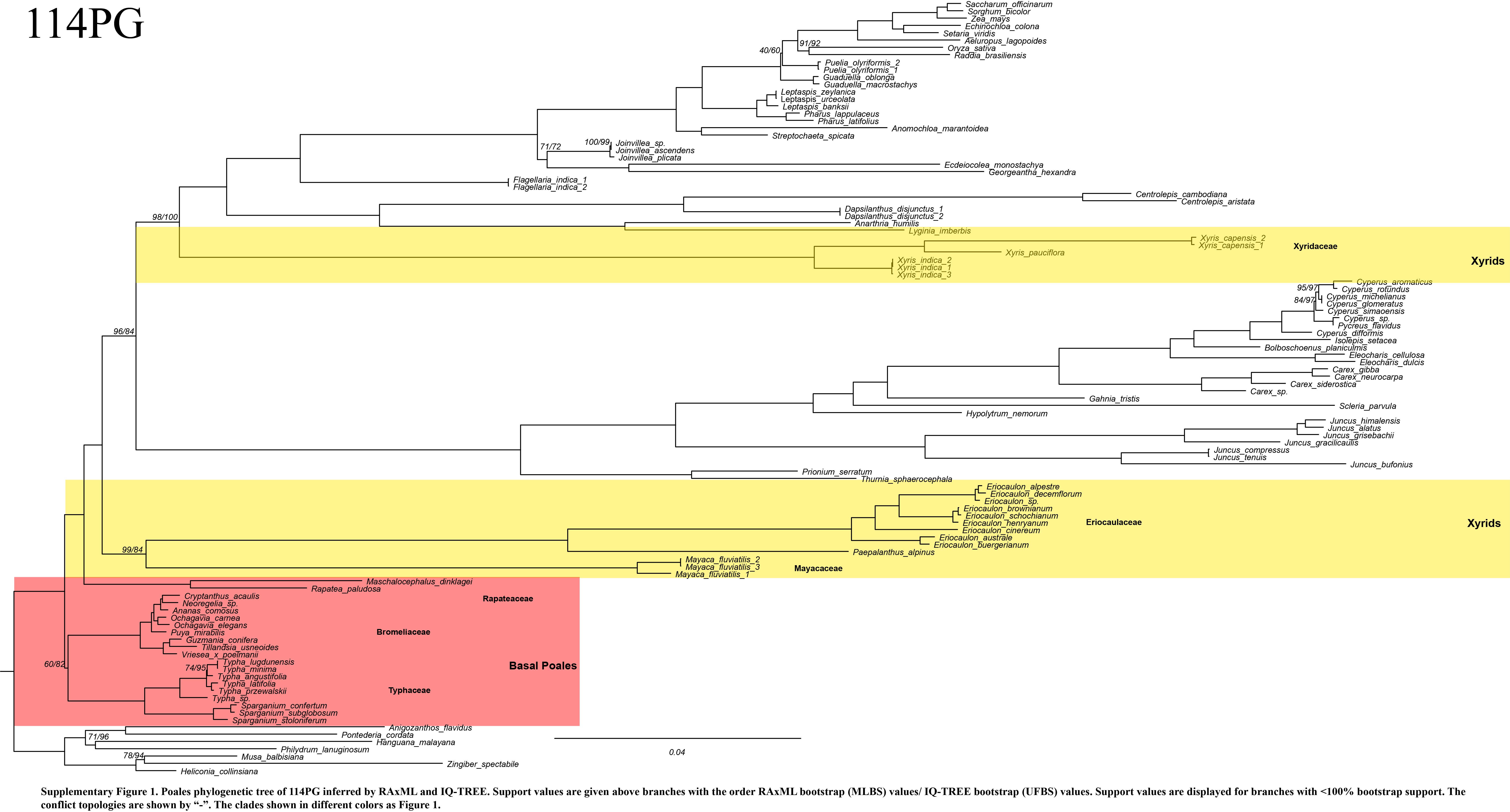

Supplement: Supplementary file 9 [file Data_Sheet_4.zip › Supplementary Figures/Supplementary Figure 1.jpg]

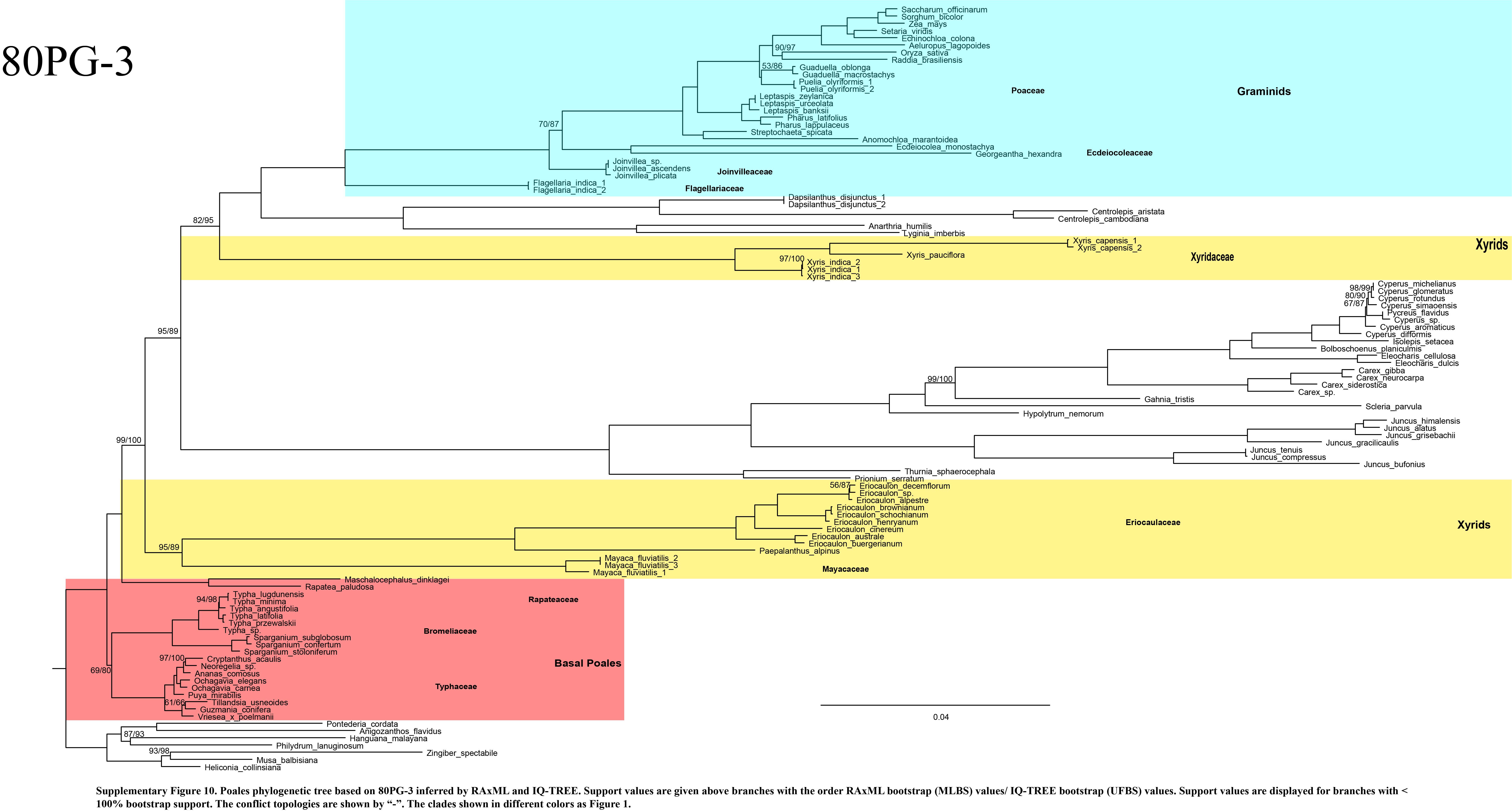

Supplement: Supplementary file 9 [file Data_Sheet_4.zip › Supplementary Figures/Supplementary Figure 10.jpg]

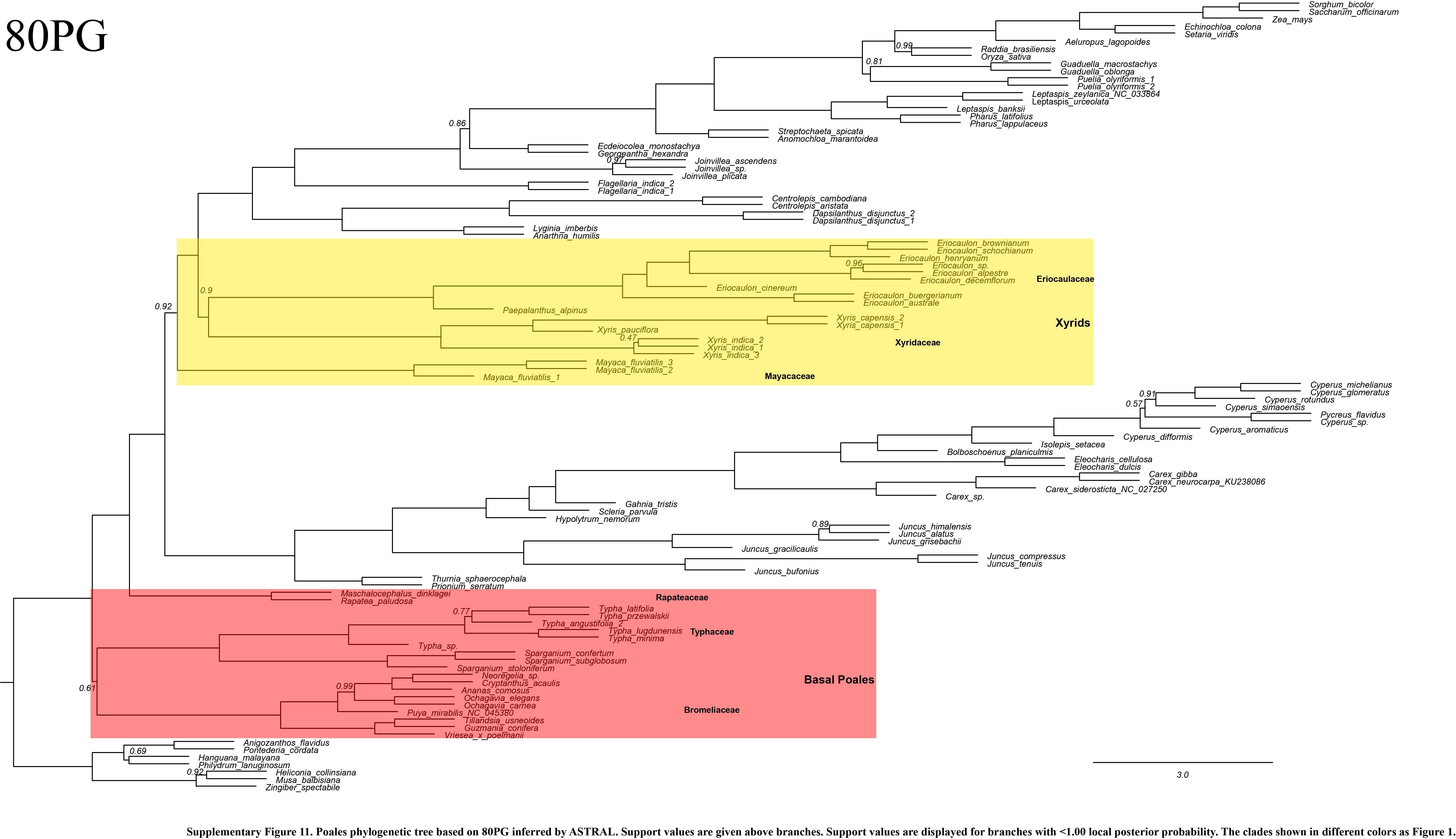

Supplement: Supplementary file 9 [file Data_Sheet_4.zip › Supplementary Figures/Supplementary Figure 11.jpg]

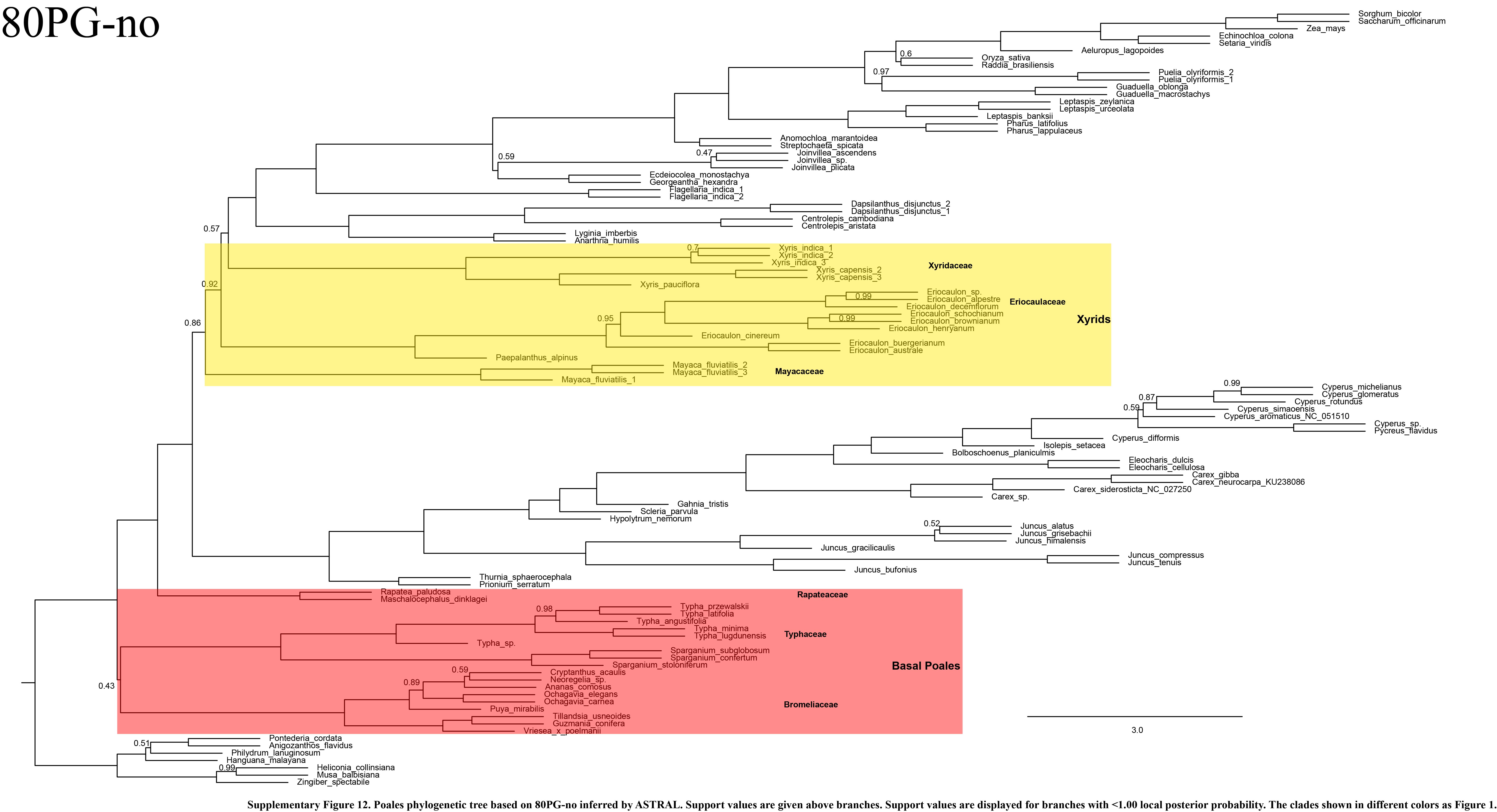

Supplement: Supplementary file 9 [file Data_Sheet_4.zip › Supplementary Figures/Supplementary Figure 12.jpg]

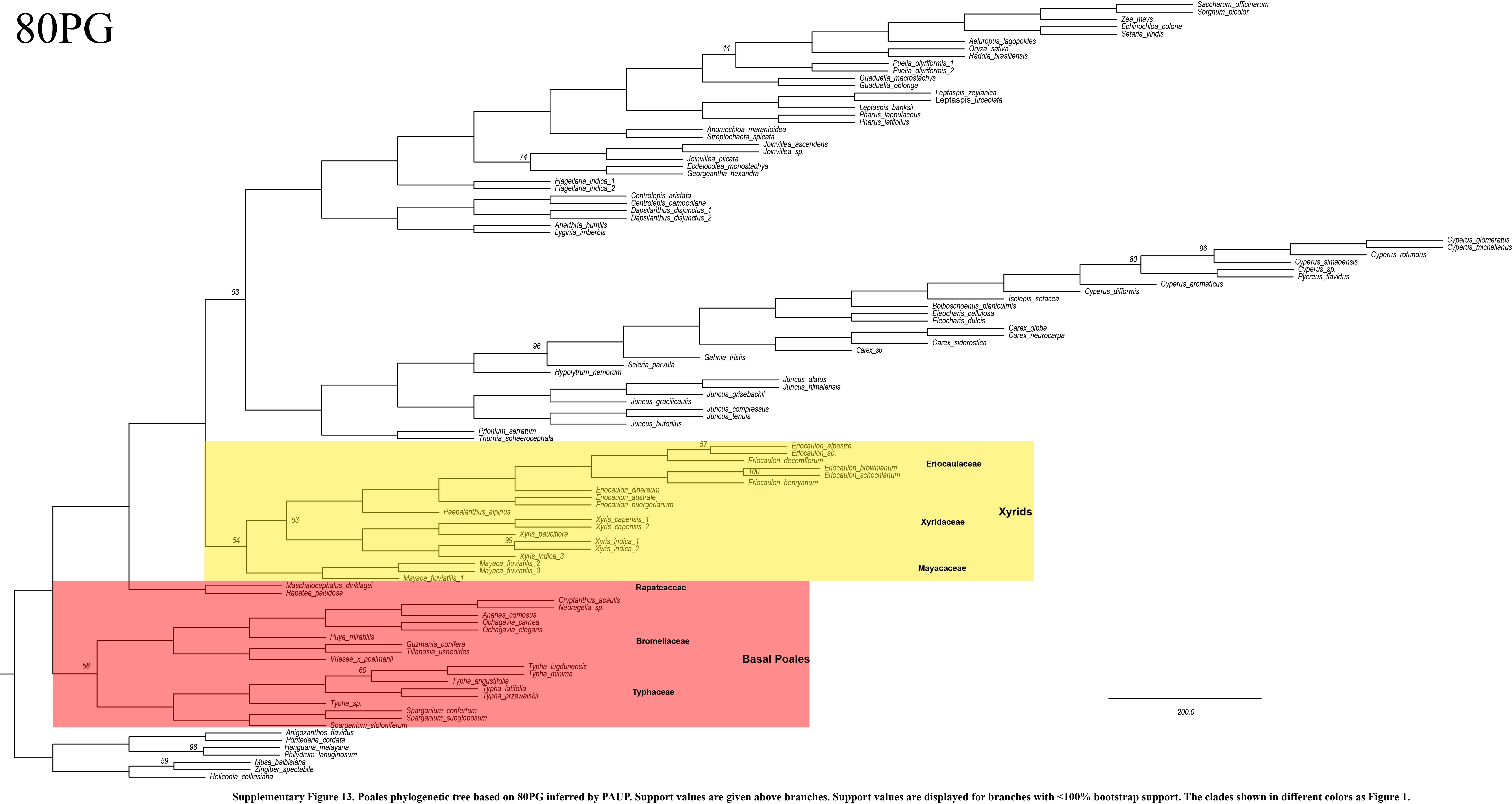

Supplement: Supplementary file 9 [file Data_Sheet_4.zip › Supplementary Figures/Supplementary Figure 13.jpg]

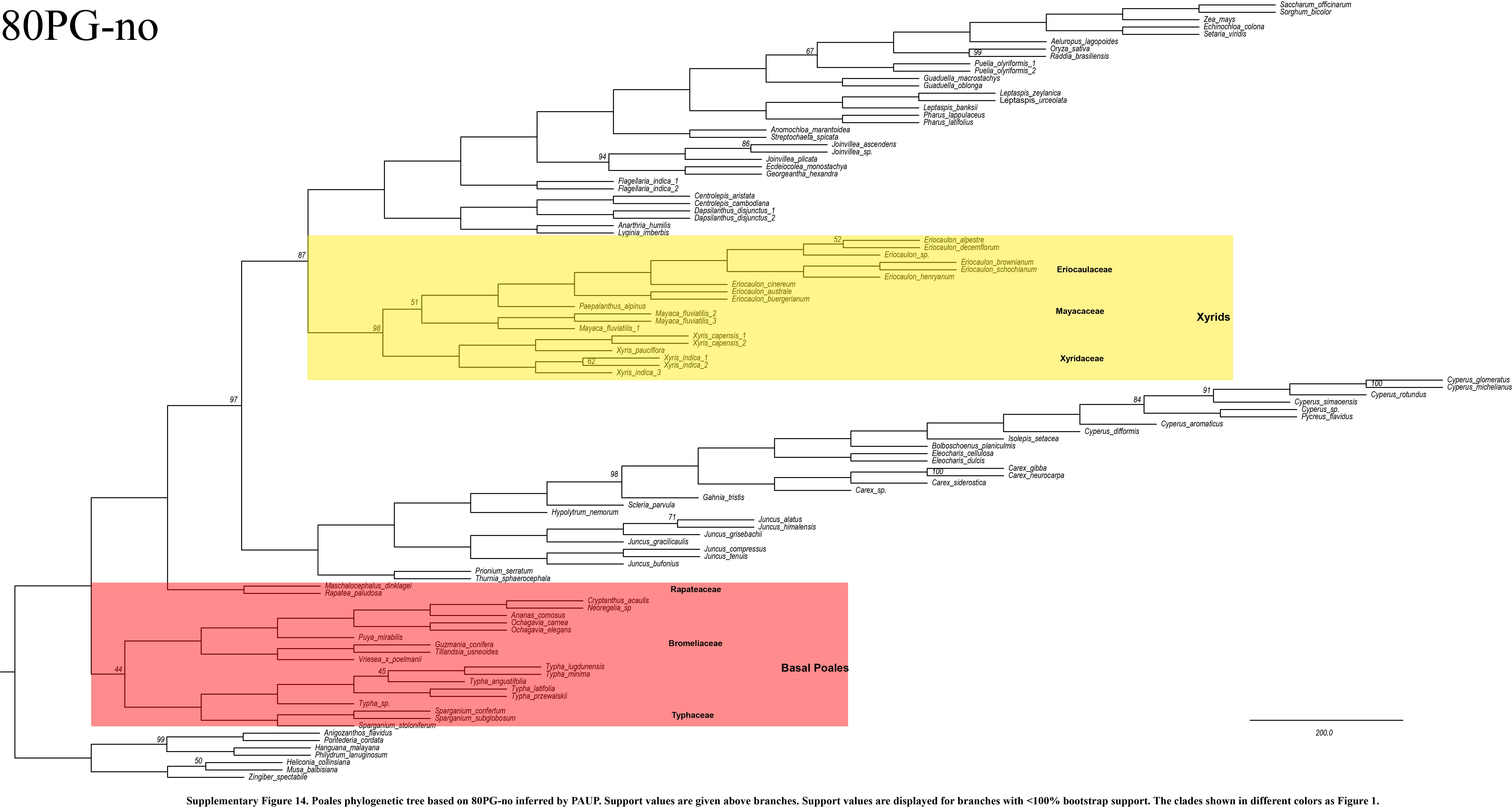

Supplement: Supplementary file 9 [file Data_Sheet_4.zip › Supplementary Figures/Supplementary Figure 14.jpg]

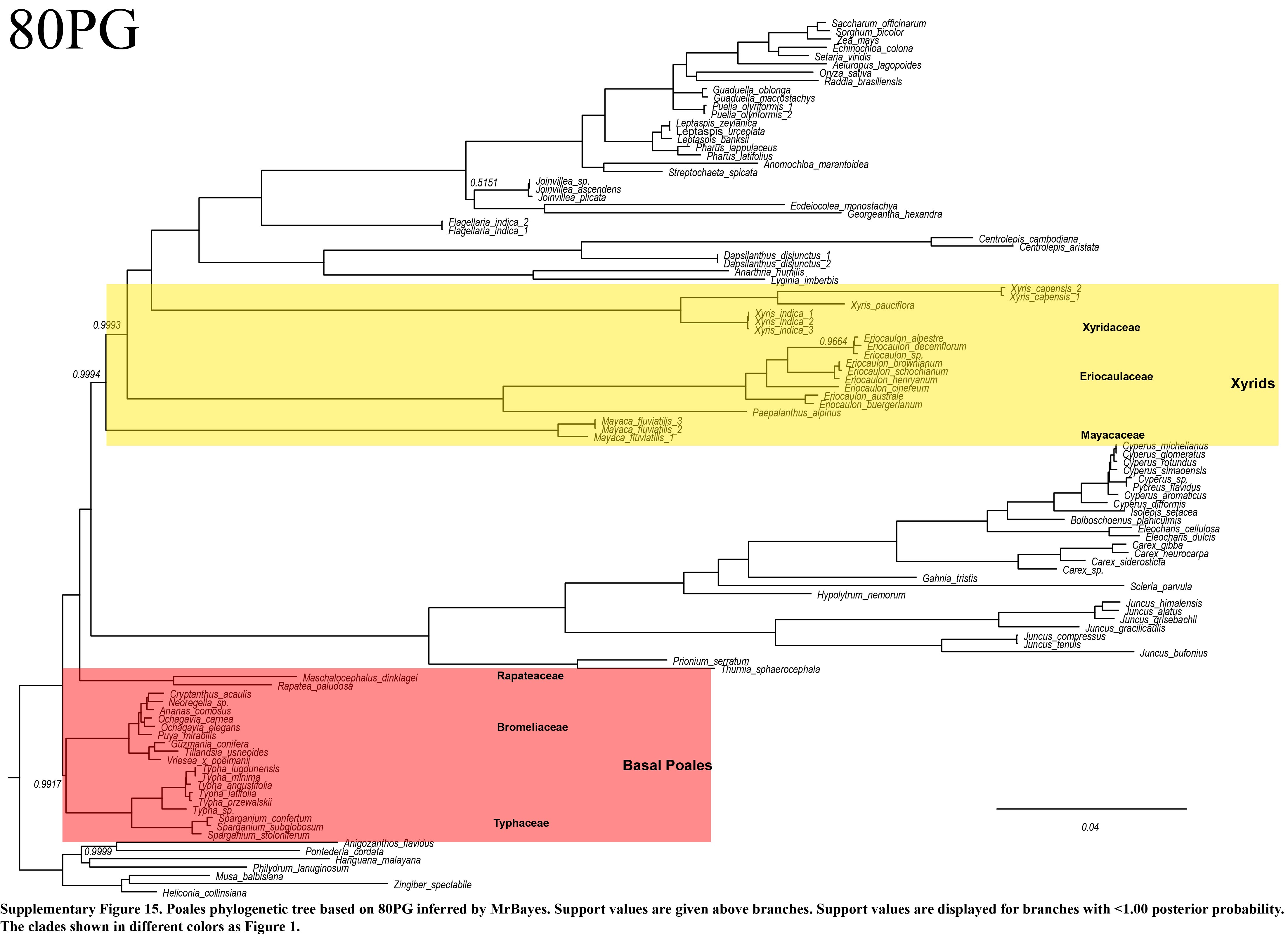

Supplement: Supplementary file 9 [file Data_Sheet_4.zip › Supplementary Figures/Supplementary Figure 15.jpg]

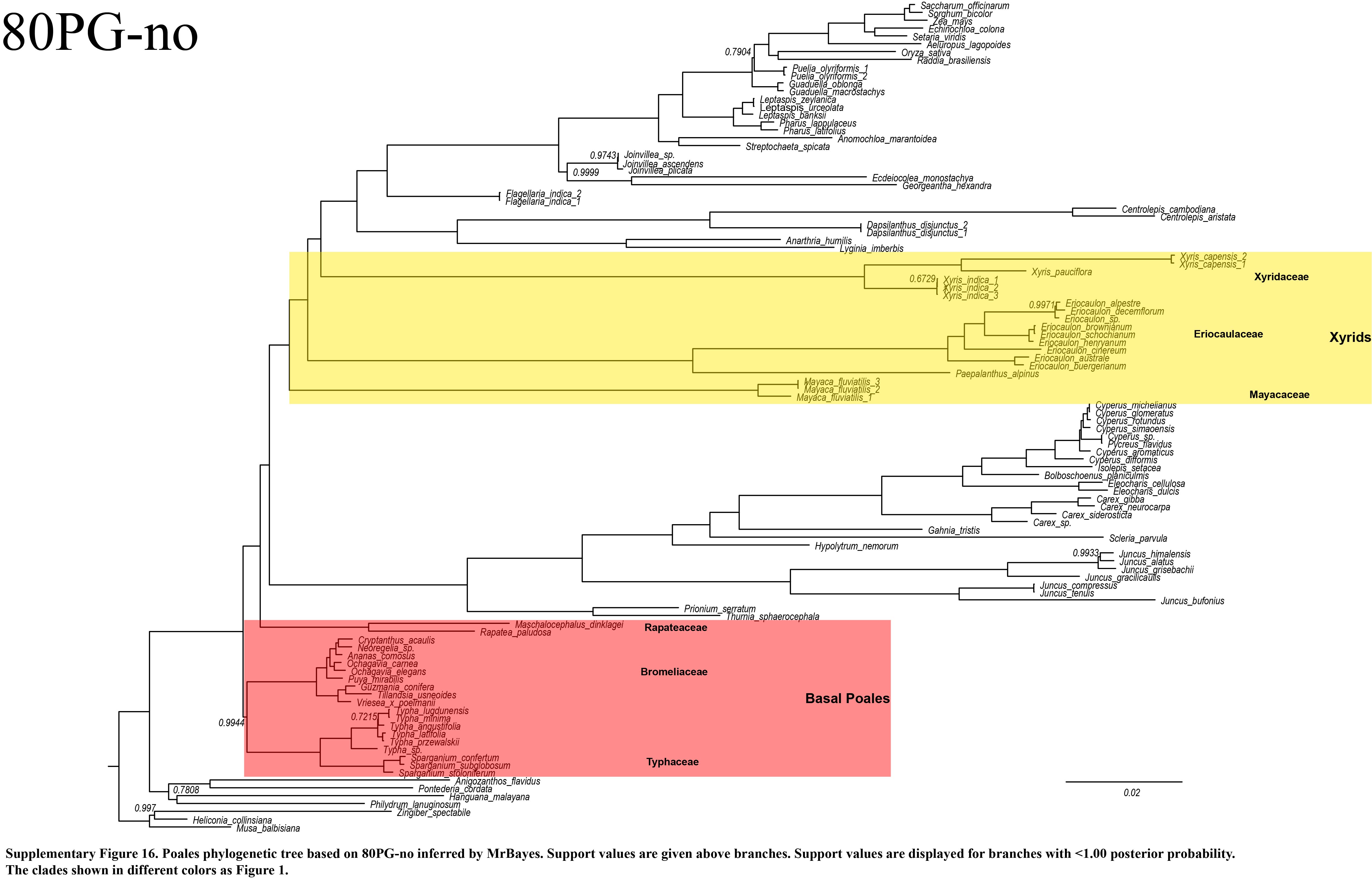

Supplement: Supplementary file 9 [file Data_Sheet_4.zip › Supplementary Figures/Supplementary Figure 16.jpg]

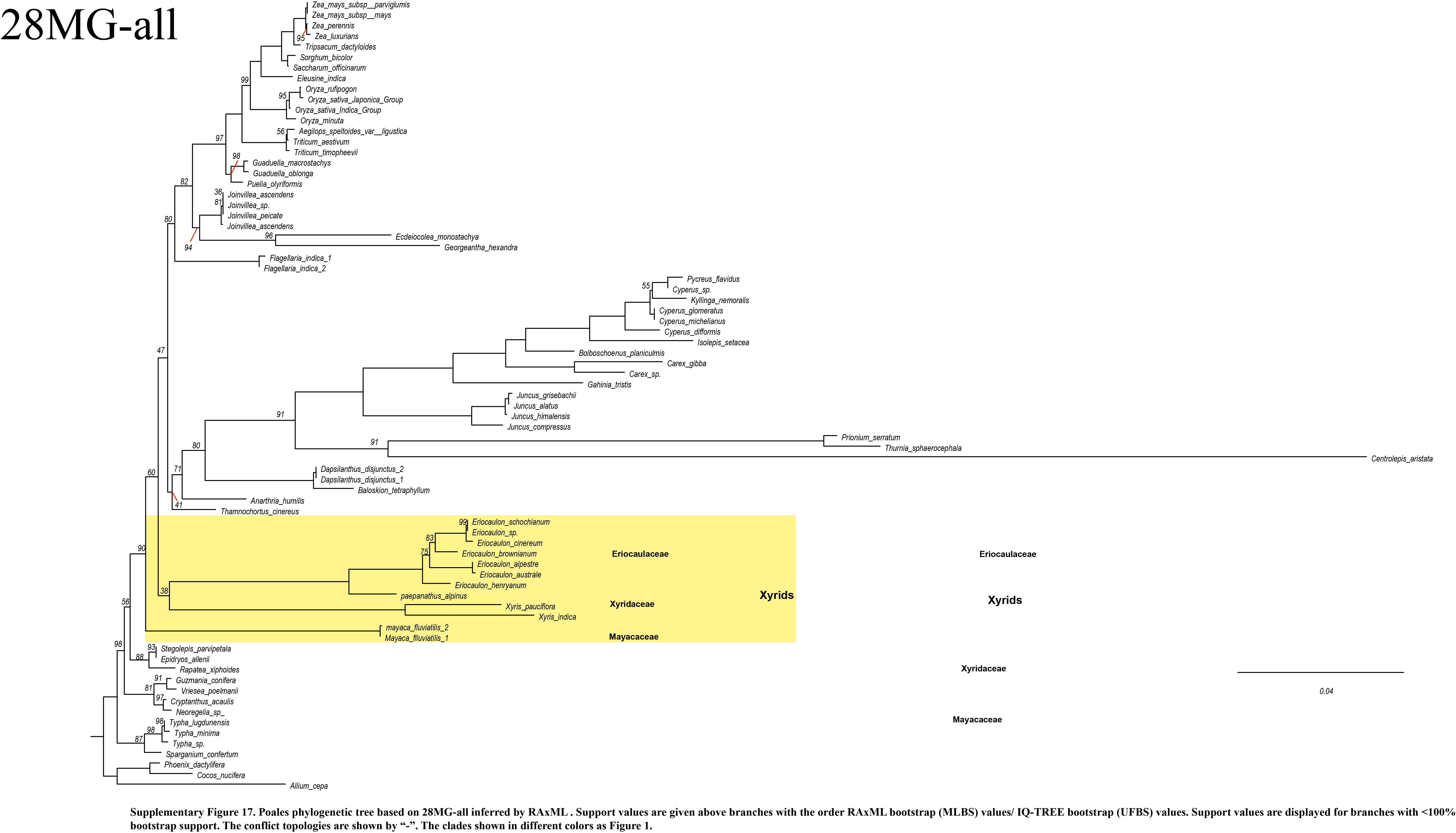

Supplement: Supplementary file 9 [file Data_Sheet_4.zip › Supplementary Figures/Supplementary Figure 17.jpg]

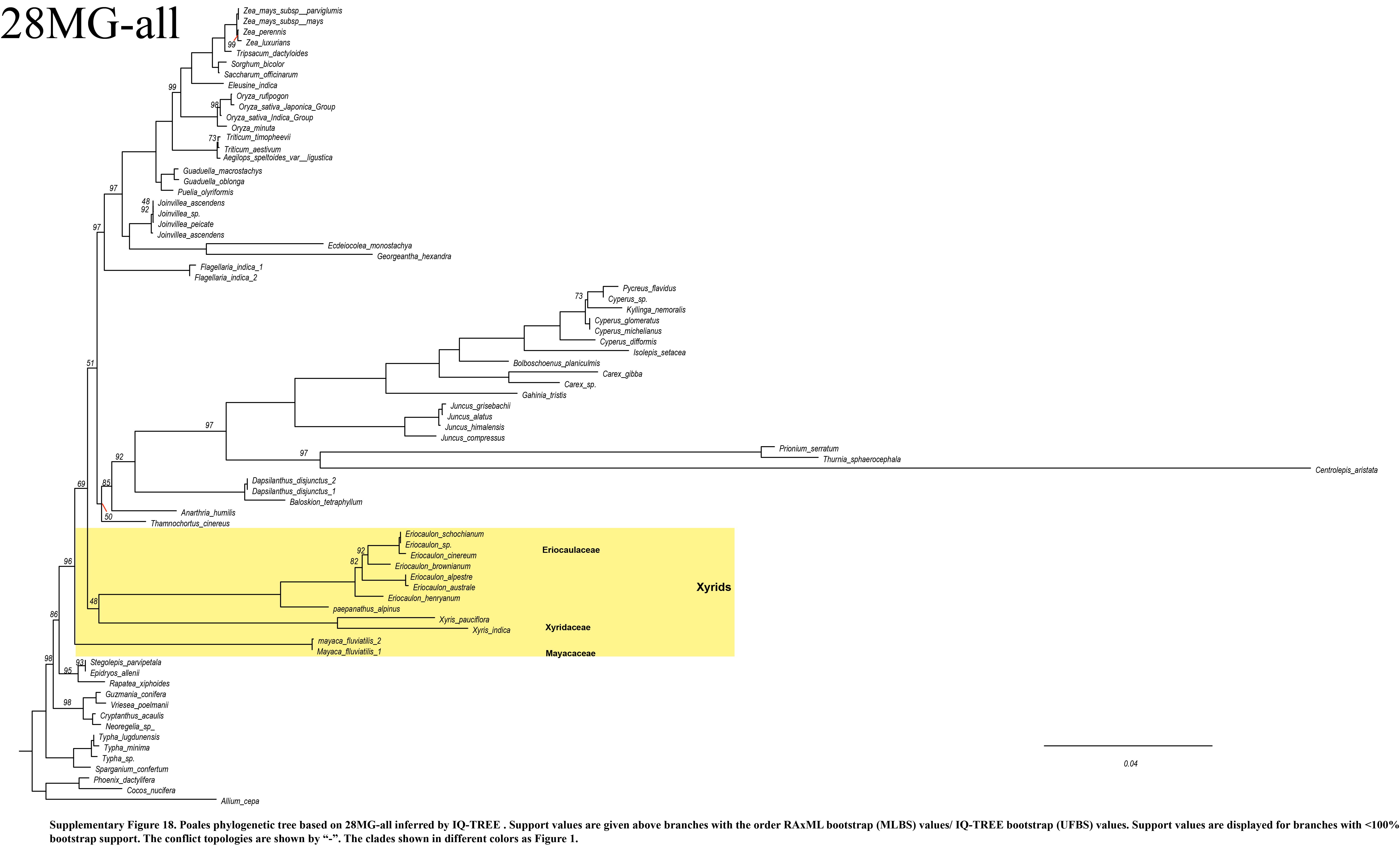

Supplement: Supplementary file 9 [file Data_Sheet_4.zip › Supplementary Figures/Supplementary Figure 18.jpg]

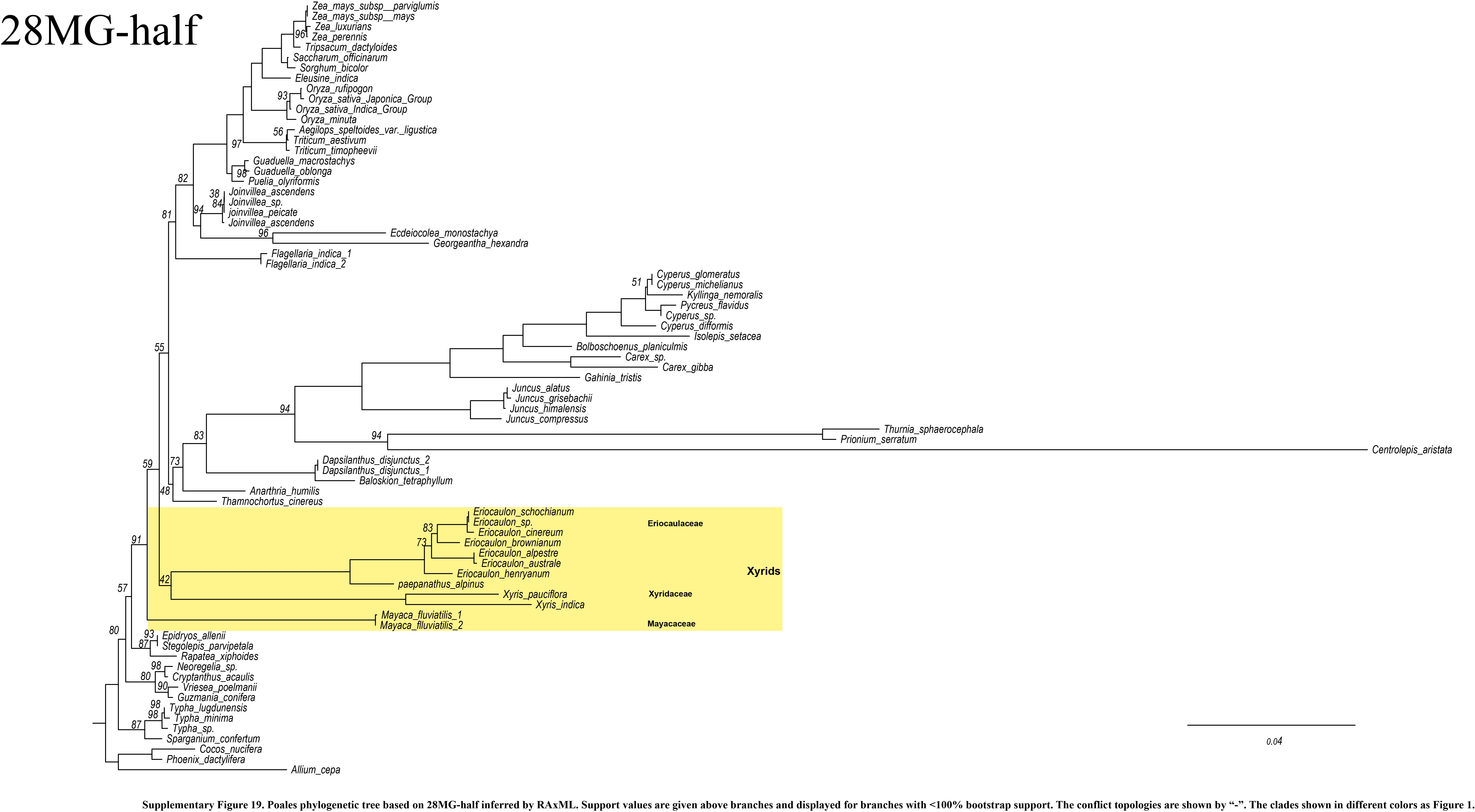

Supplement: Supplementary file 9 [file Data_Sheet_4.zip › Supplementary Figures/Supplementary Figure 19.jpg]

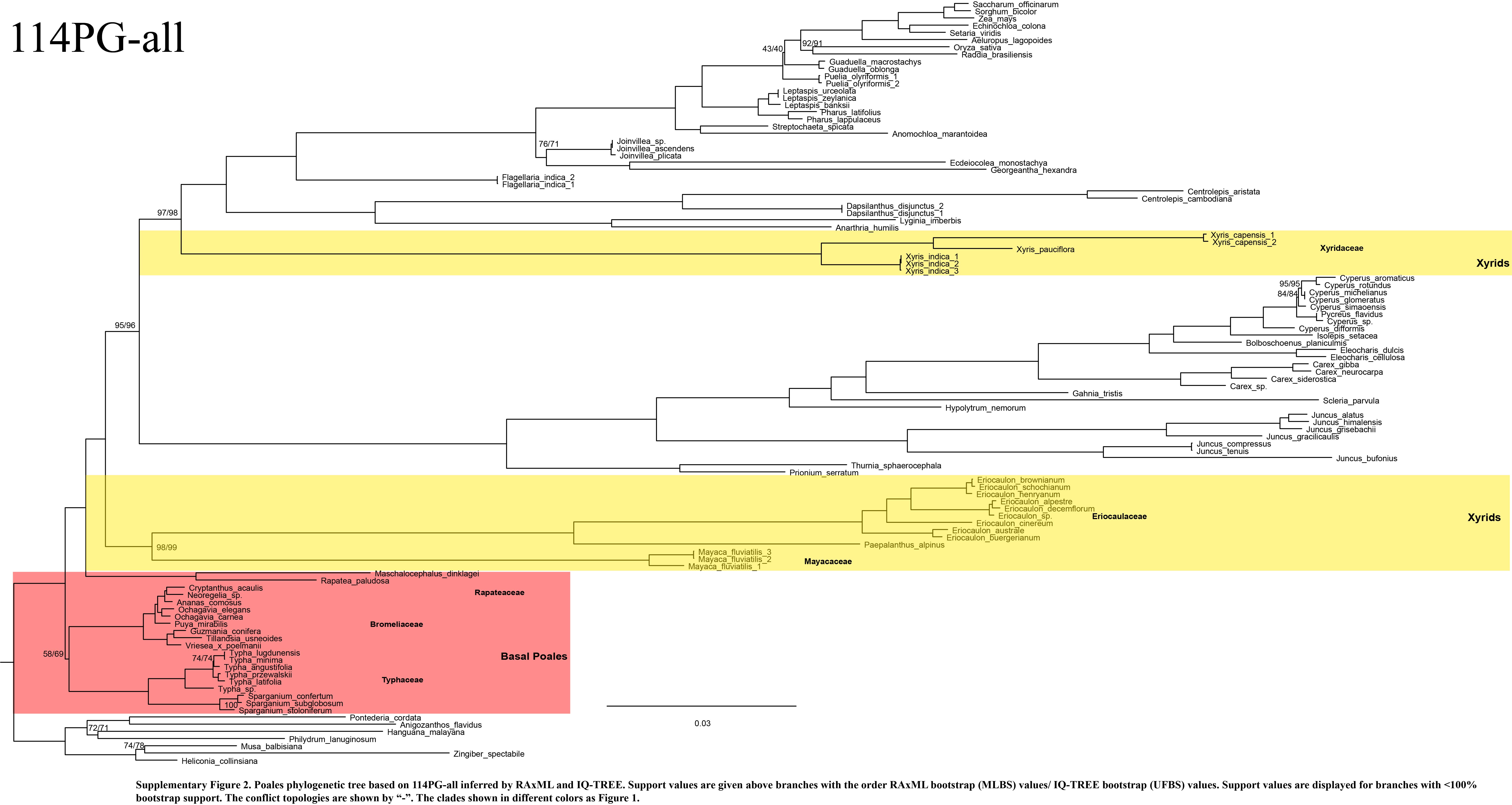

Supplement: Supplementary file 9 [file Data_Sheet_4.zip › Supplementary Figures/Supplementary Figure 2.jpg]

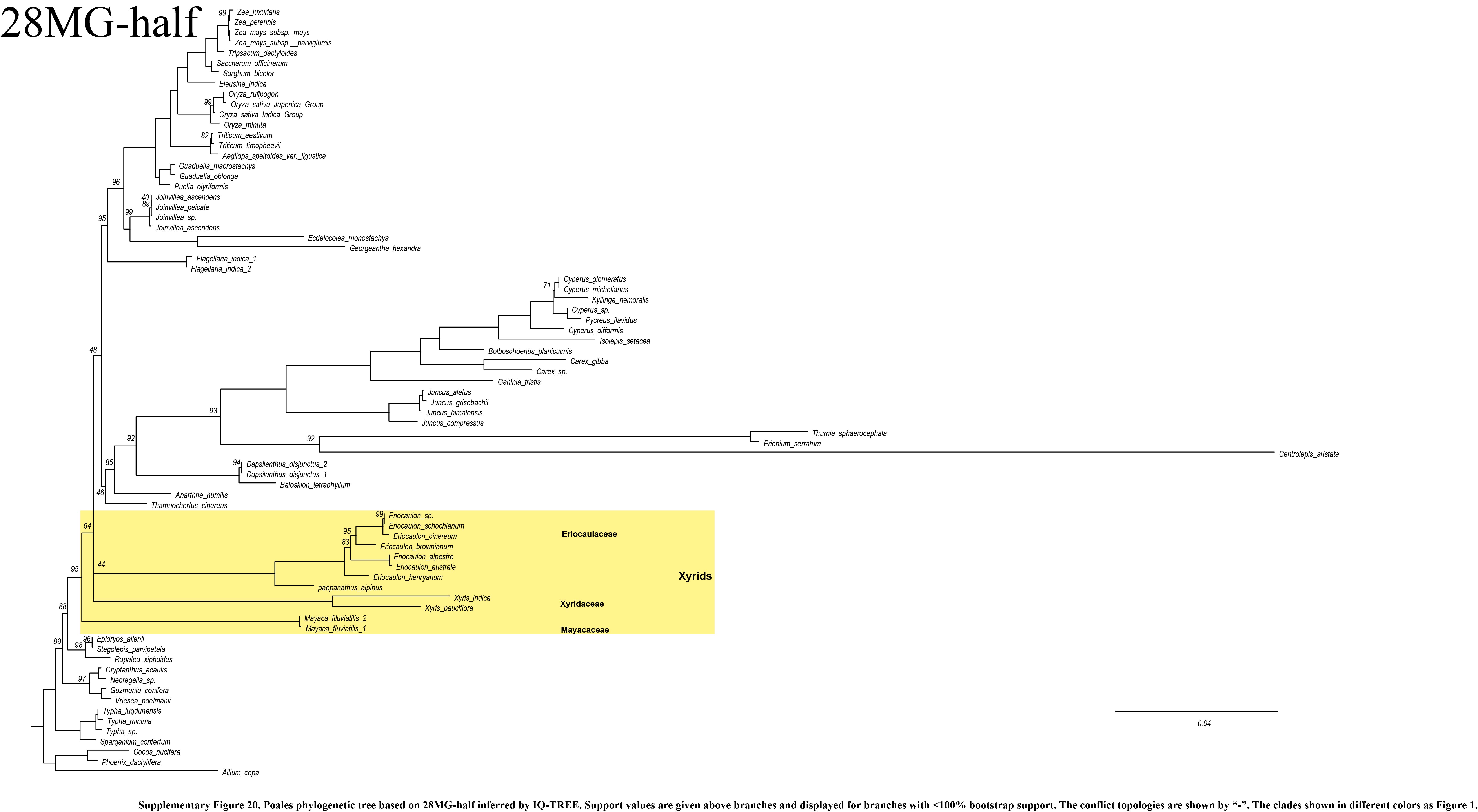

Supplement: Supplementary file 9 [file Data_Sheet_4.zip › Supplementary Figures/Supplementary Figure 20.jpg]

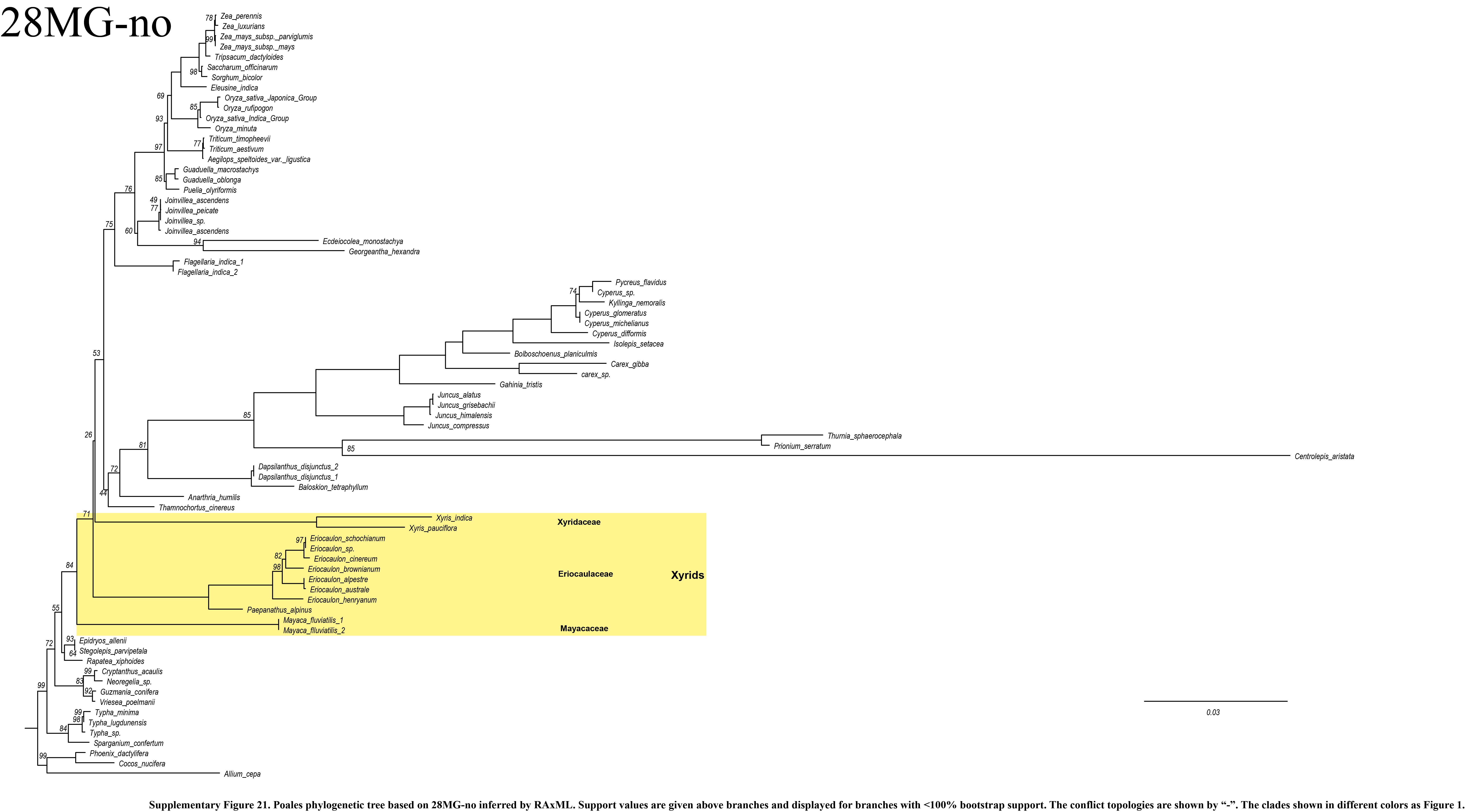

Supplement: Supplementary file 9 [file Data_Sheet_4.zip › Supplementary Figures/Supplementary Figure 21.jpg]

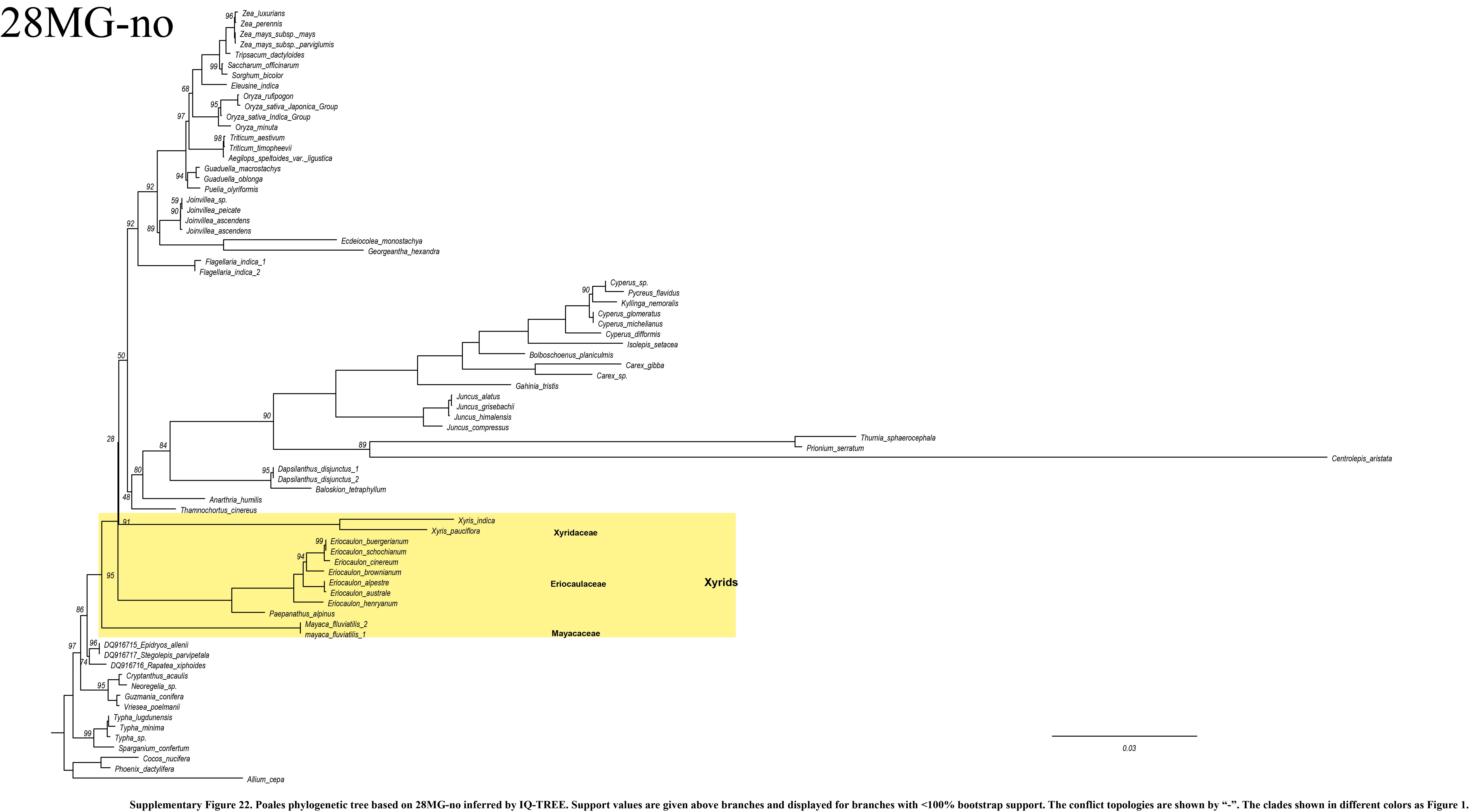

Supplement: Supplementary file 9 [file Data_Sheet_4.zip › Supplementary Figures/Supplementary Figure 22.jpg]

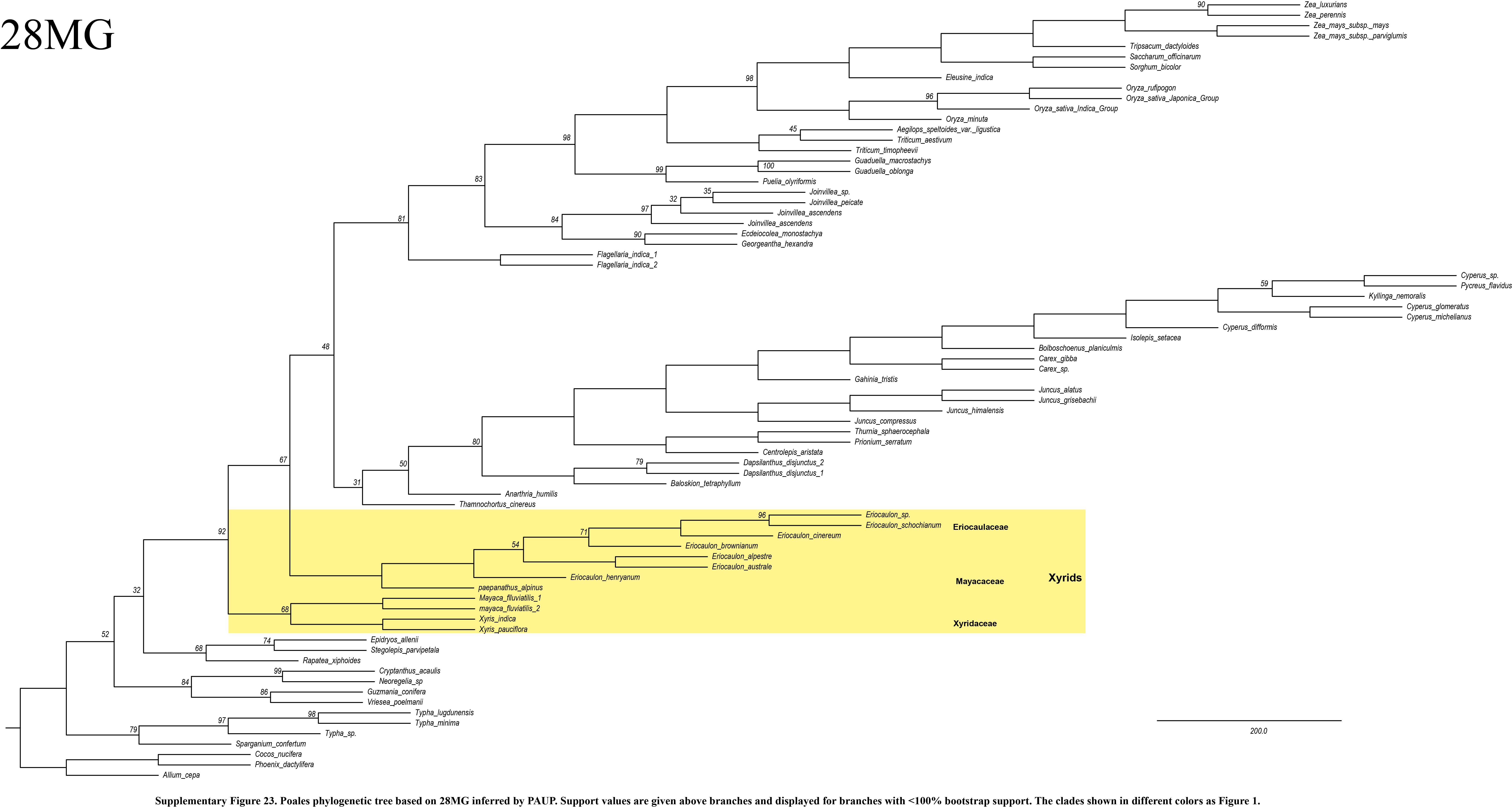

Supplement: Supplementary file 9 [file Data_Sheet_4.zip › Supplementary Figures/Supplementary Figure 23.jpg]

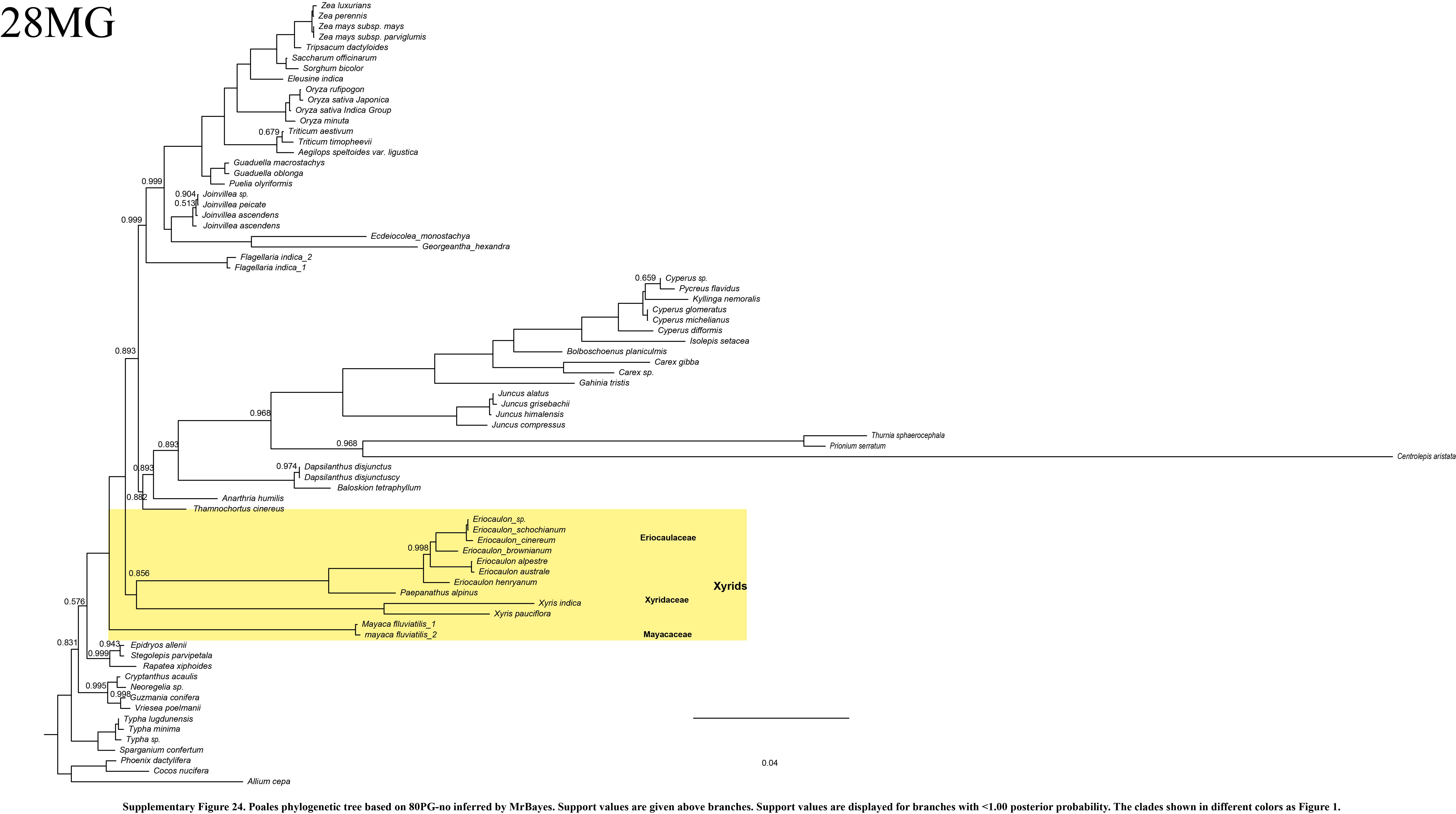

Supplement: Supplementary file 9 [file Data_Sheet_4.zip › Supplementary Figures/Supplementary Figure 24.jpg]

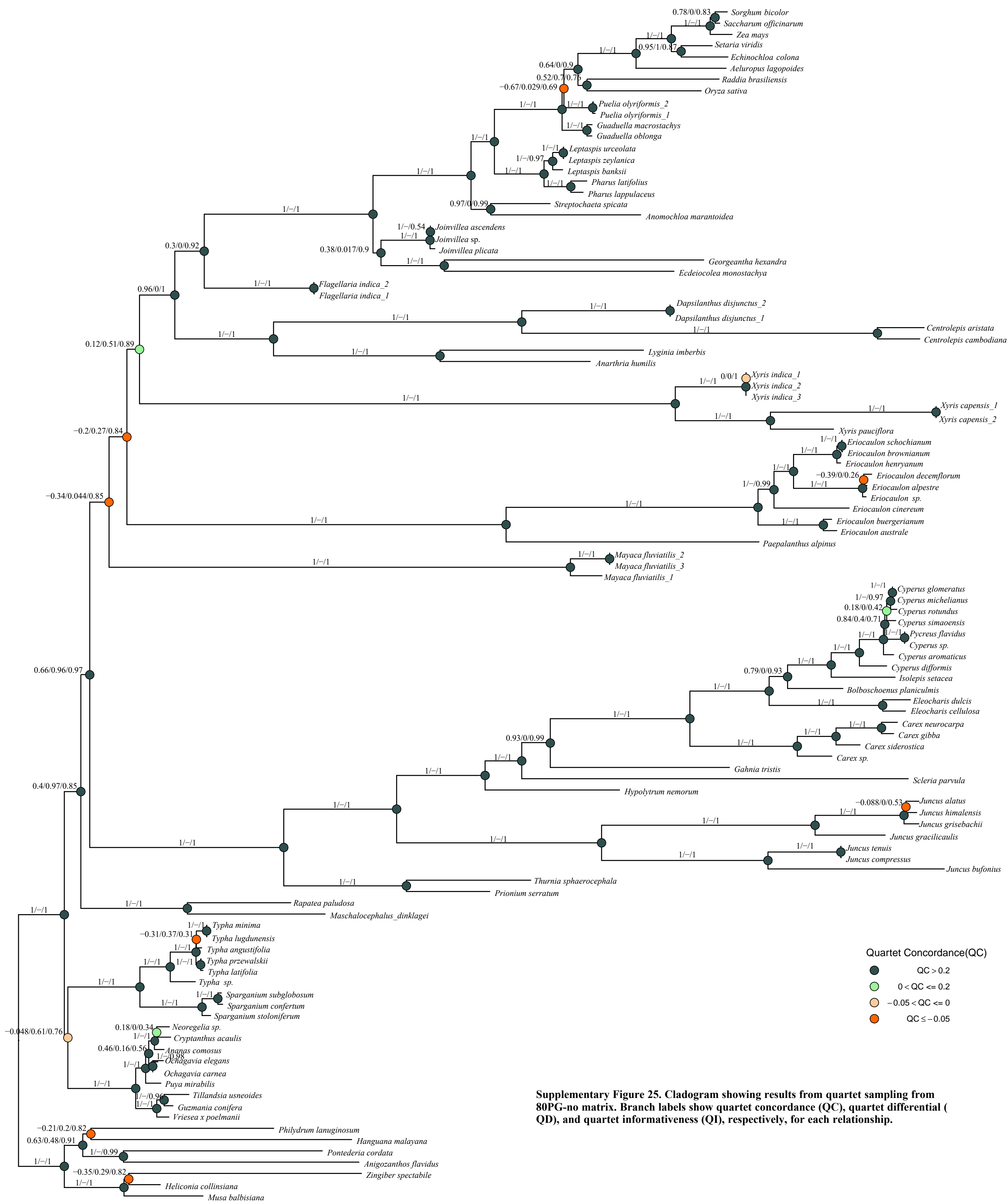

Supplement: Supplementary file 9 [file Data_Sheet_4.zip › Supplementary Figures/Supplementary Figure 25.pdf]

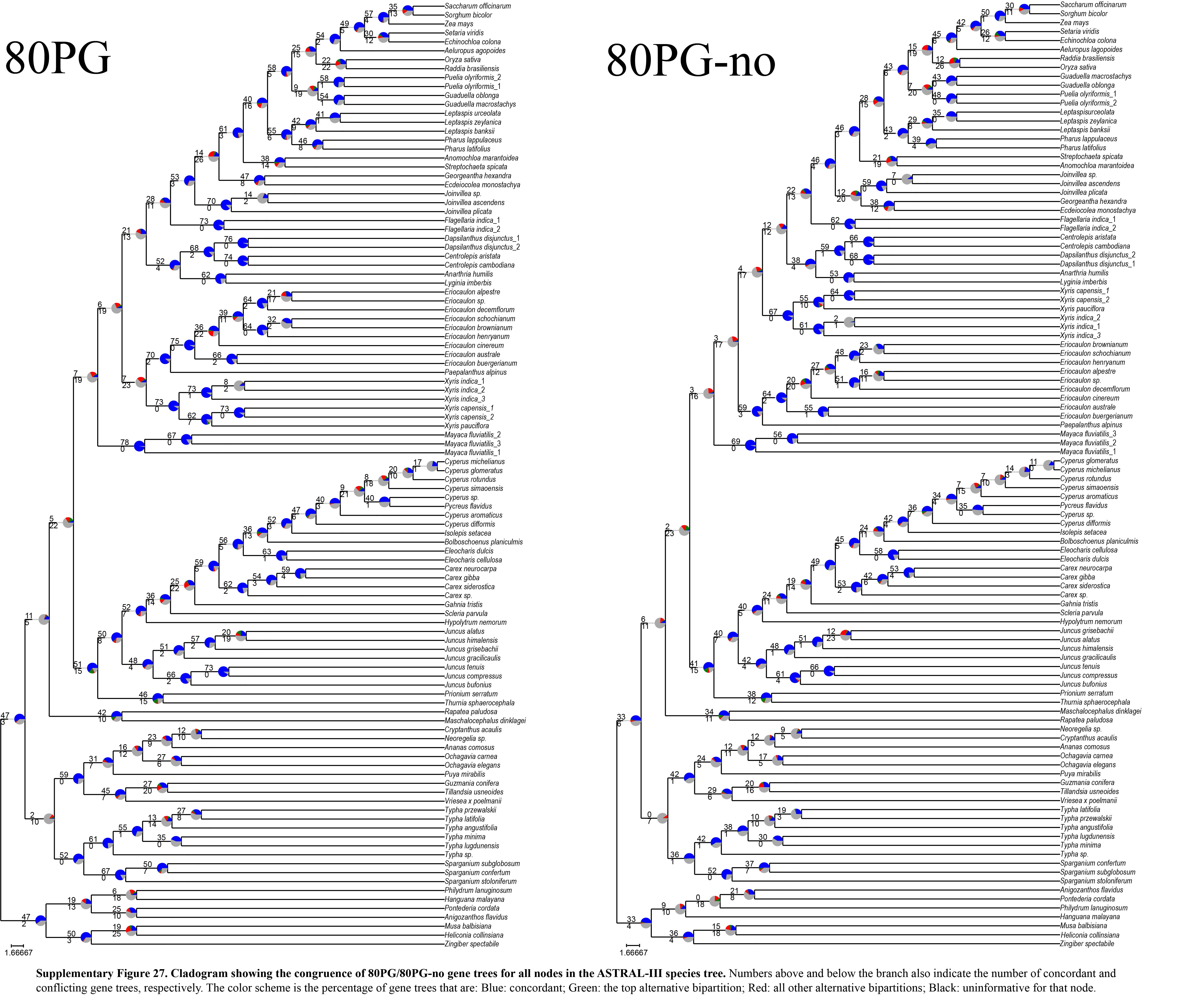

Supplement: Supplementary file 9 [file Data_Sheet_4.zip › Supplementary Figures/Supplementary Figure 27.jpg]

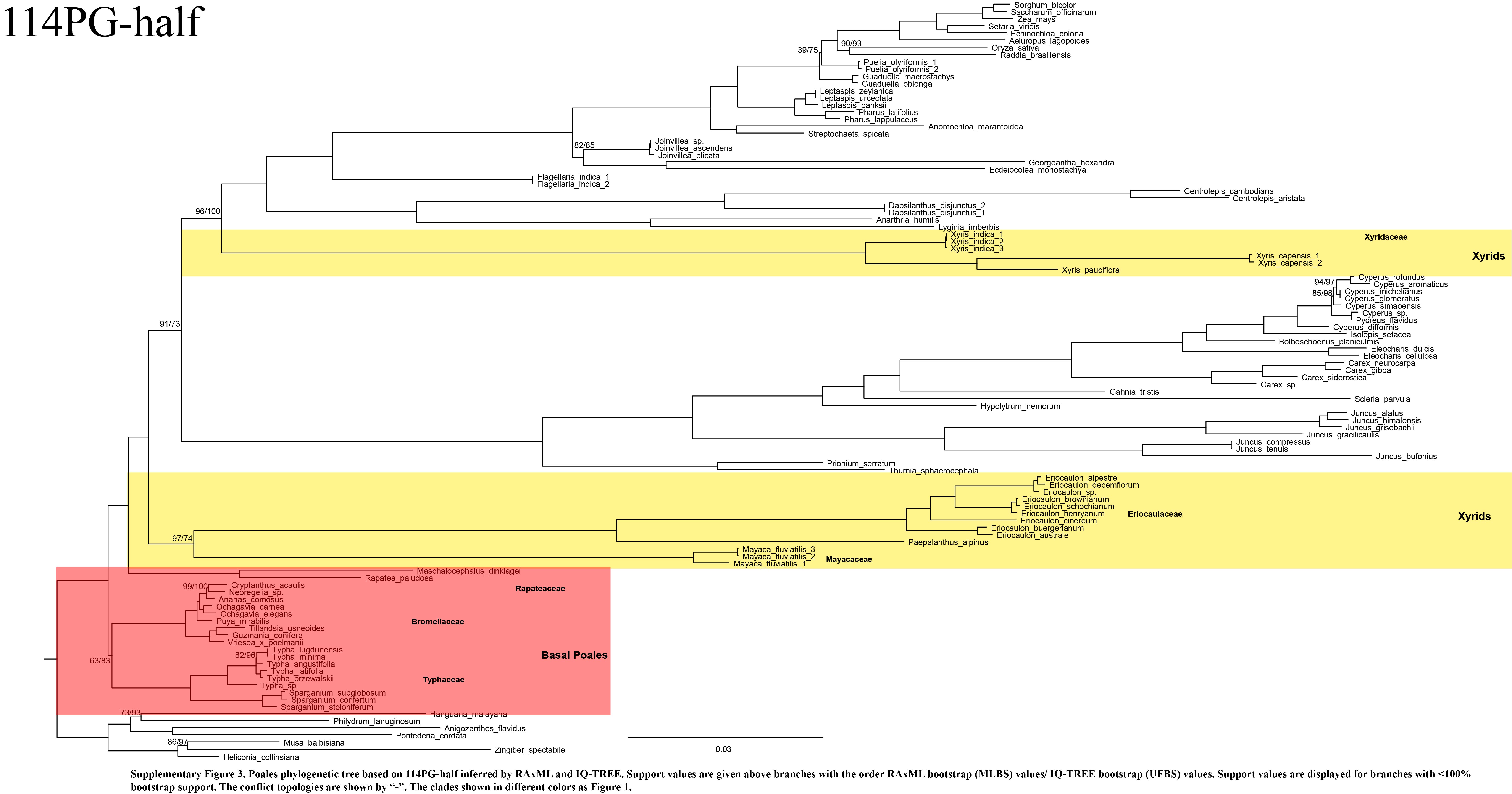

Supplement: Supplementary file 9 [file Data_Sheet_4.zip › Supplementary Figures/Supplementary Figure 3.jpg]

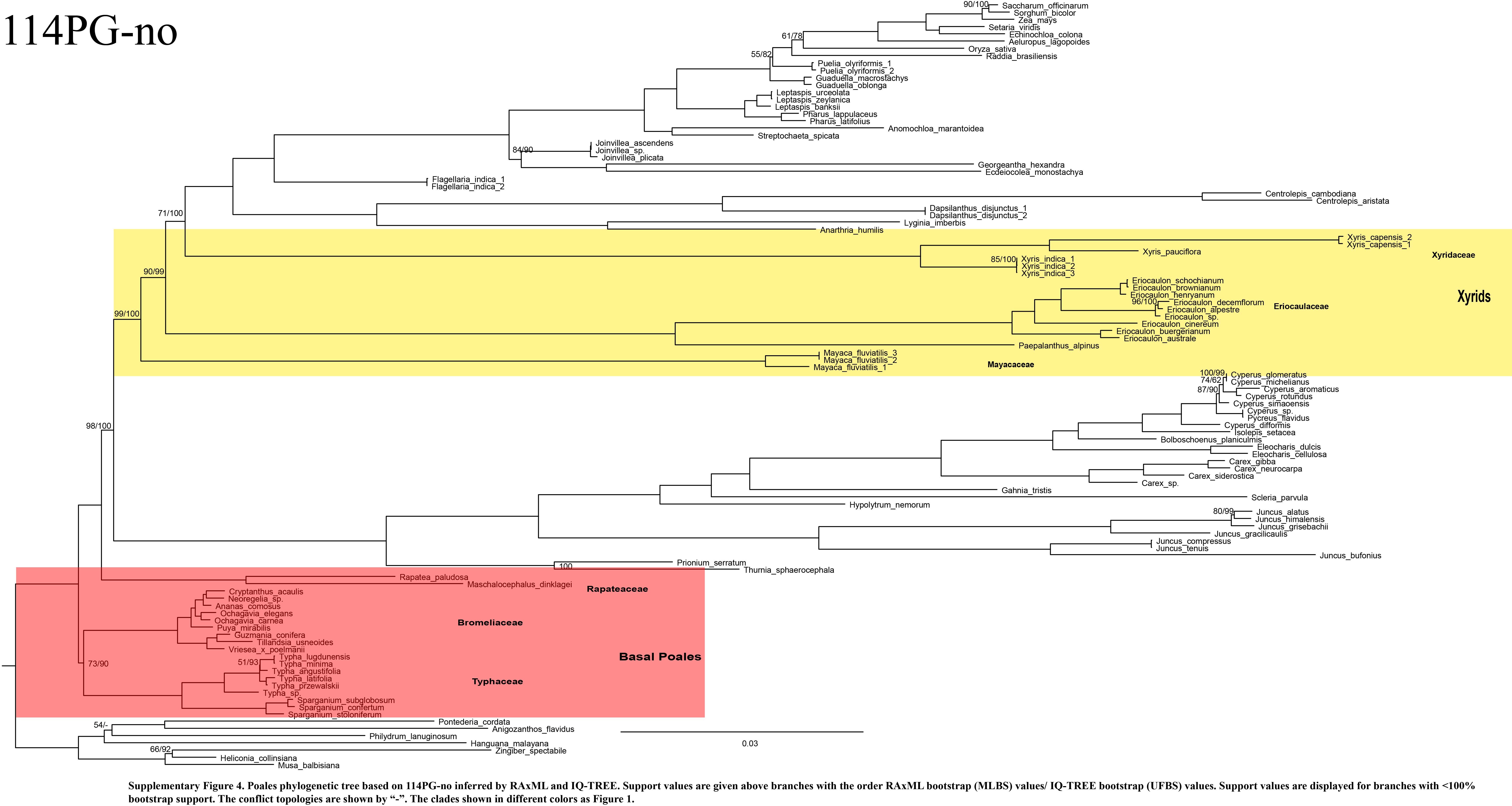

Supplement: Supplementary file 9 [file Data_Sheet_4.zip › Supplementary Figures/Supplementary Figure 4.jpg]

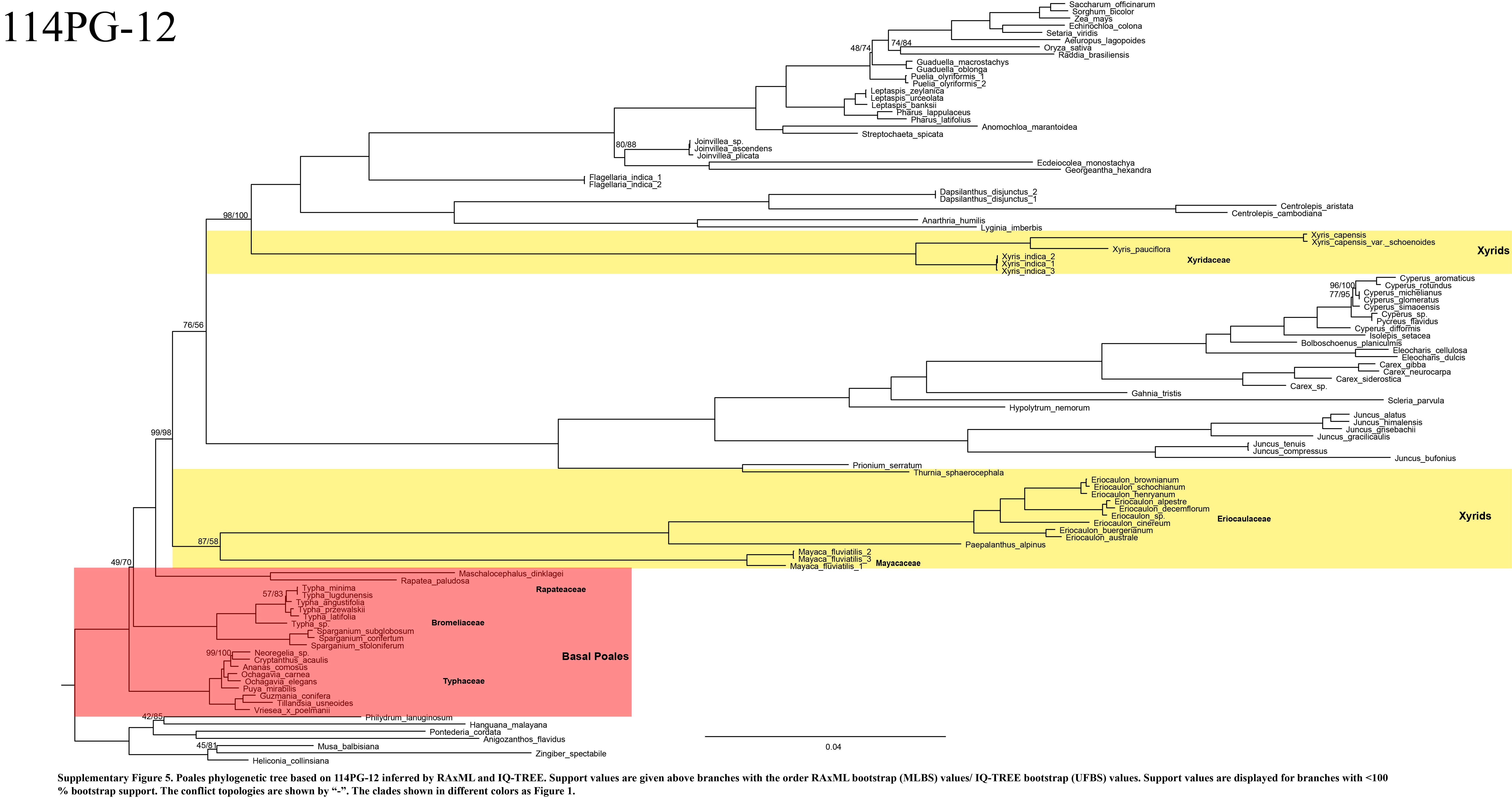

Supplement: Supplementary file 9 [file Data_Sheet_4.zip › Supplementary Figures/Supplementary Figure 5.jpg]

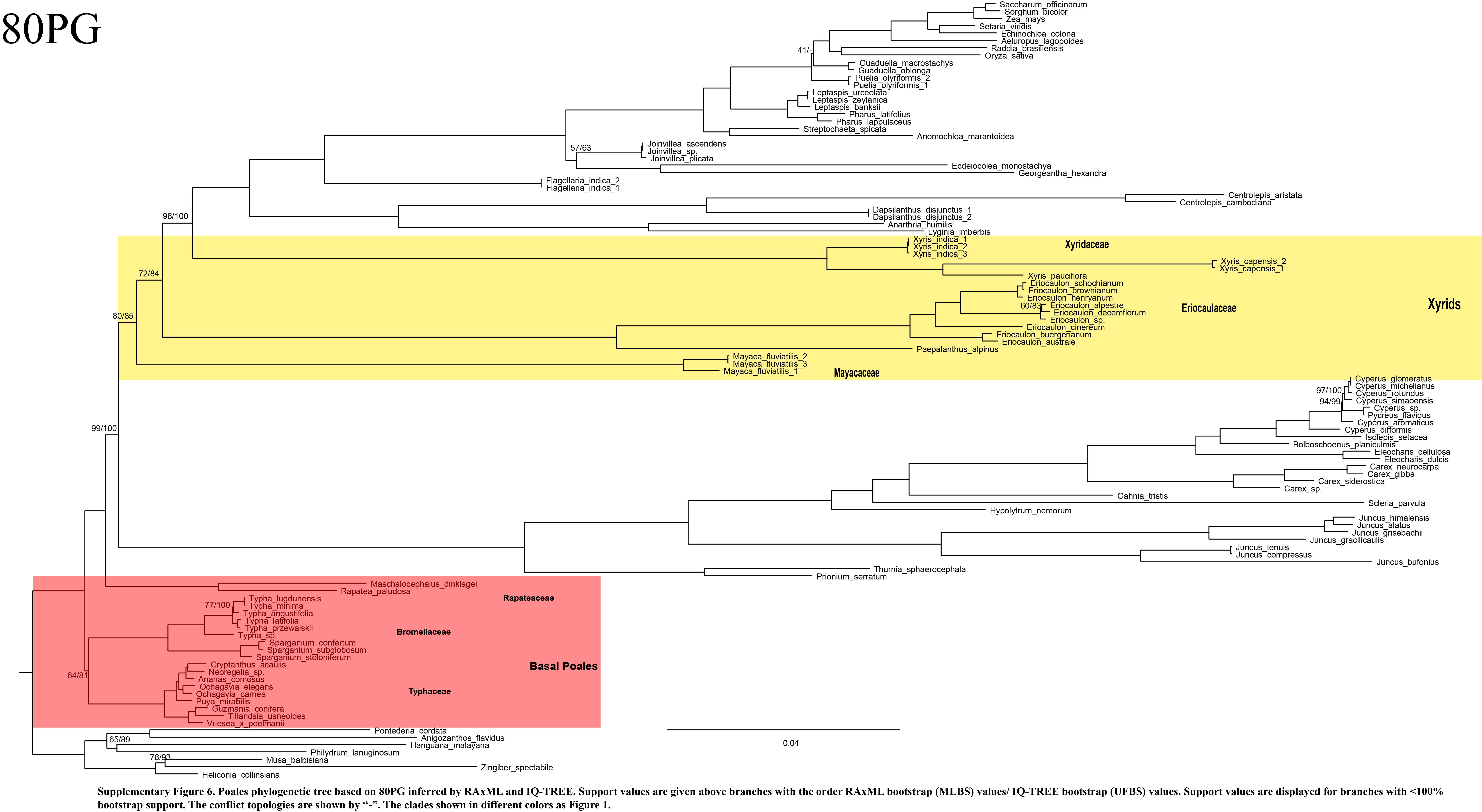

Supplement: Supplementary file 9 [file Data_Sheet_4.zip › Supplementary Figures/Supplementary Figure 6.jpg]

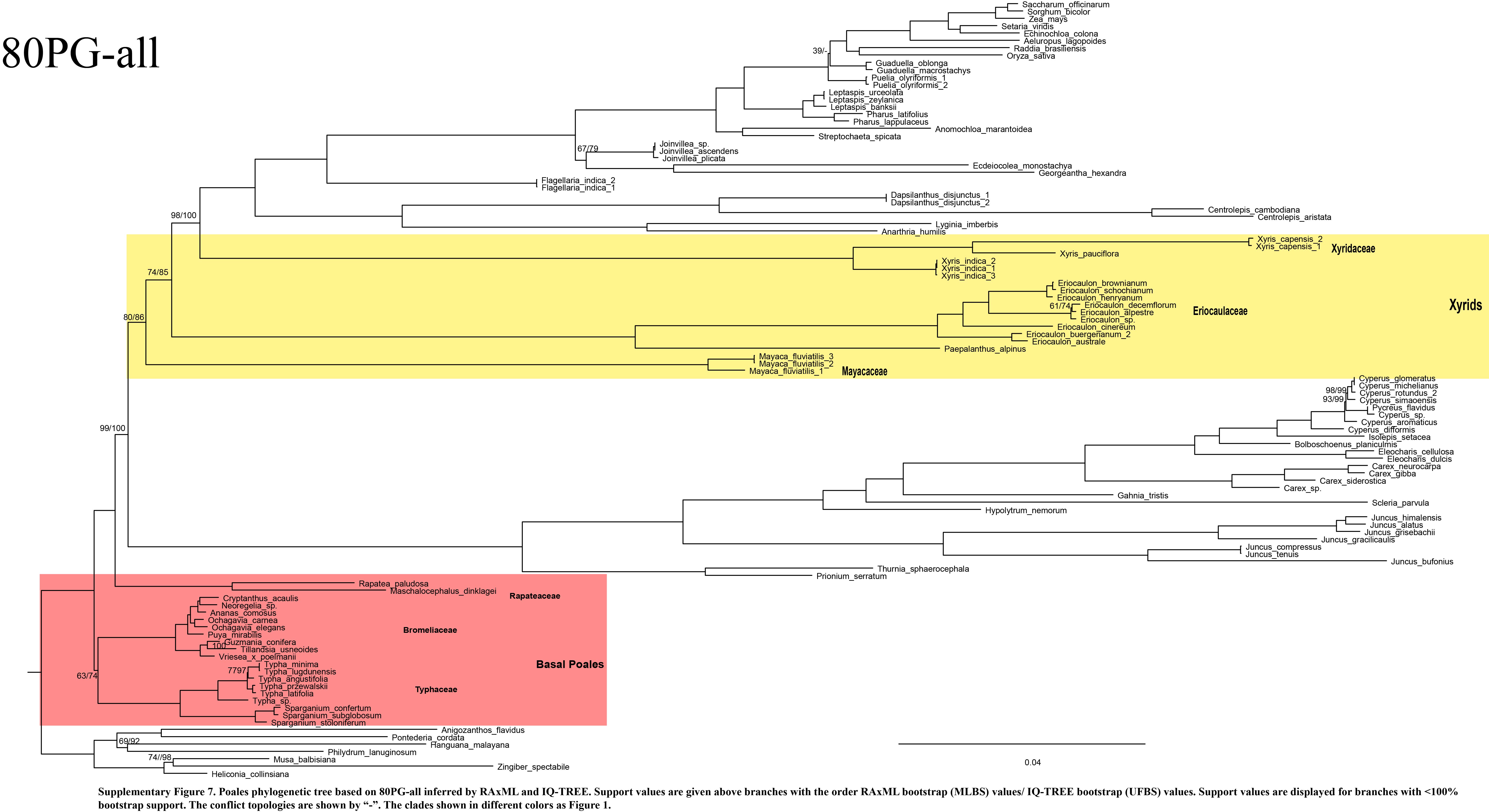

Supplement: Supplementary file 9 [file Data_Sheet_4.zip › Supplementary Figures/Supplementary Figure 7.jpg]

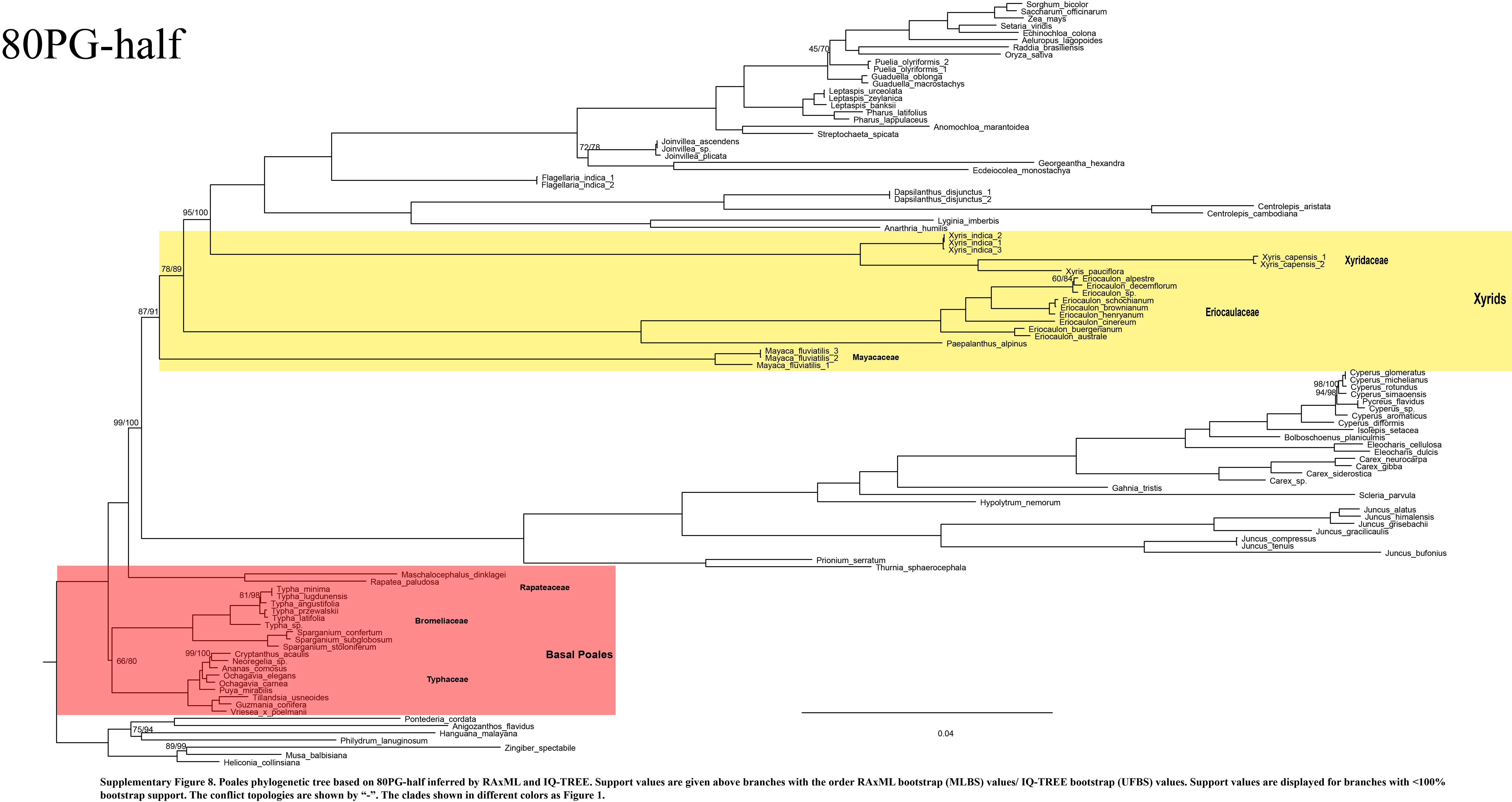

Supplement: Supplementary file 9 [file Data_Sheet_4.zip › Supplementary Figures/Supplementary Figure 8.jpg]

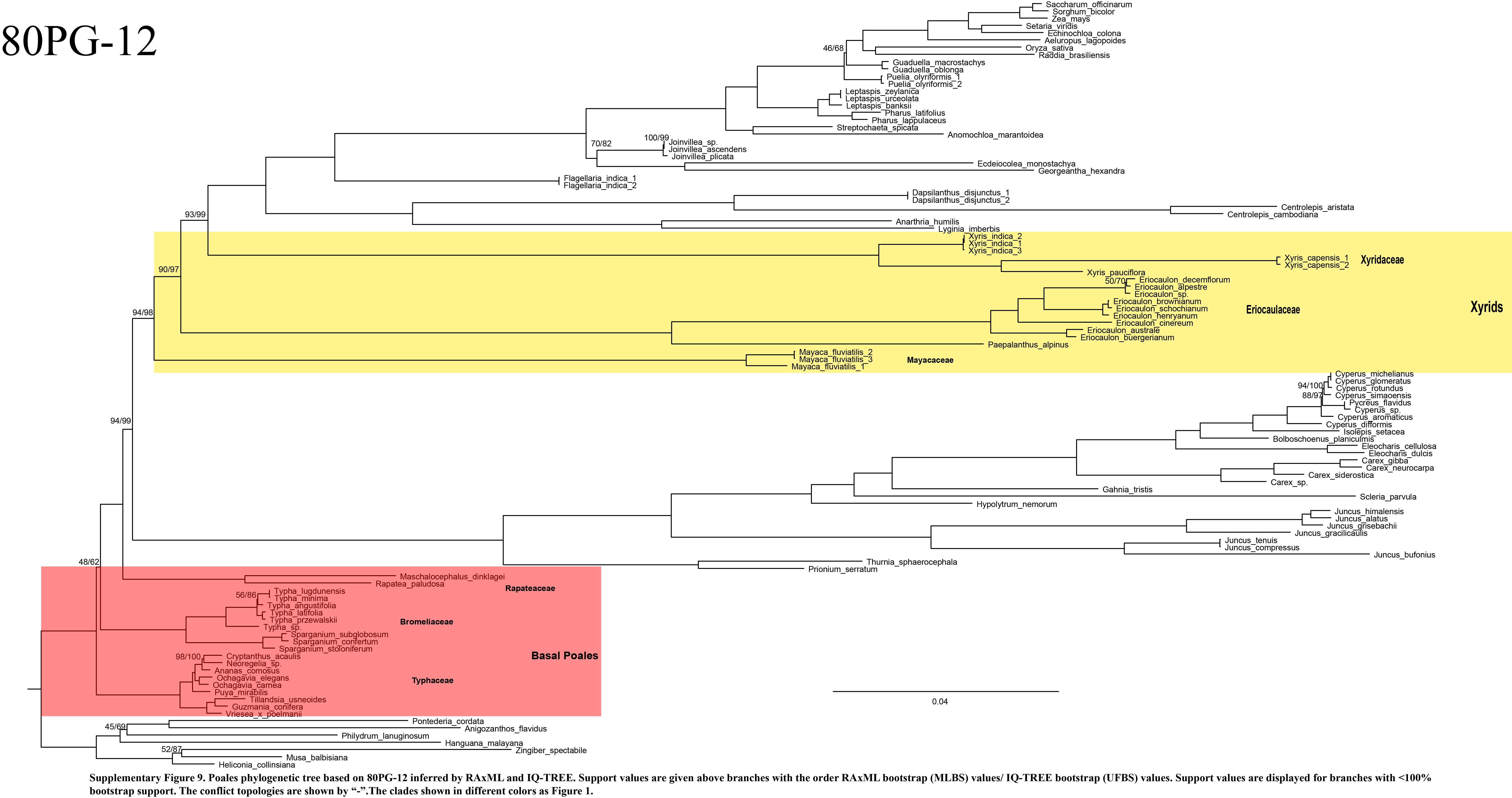

Supplement: Supplementary file 9 [file Data_Sheet_4.zip › Supplementary Figures/Supplementary Figure 9.jpg]
